# Cardiovascular, Metabolic and Endocrine, Cancer, Mortality, Derma, and Other Outcomes of Olive Oil and Oleic Acid: An Umbrella Review

**DOI:** 10.1002/fsn3.71115

**Published:** 2025-10-22

**Authors:** Xinrui Zou, Hong Liu

**Affiliations:** ^1^ Division of Surgery, Institute of Integrated Traditional and Western Medicine West China Hospital, Sichuan University Chengdu China

**Keywords:** anthropometric indices, cancer, monounsaturated fatty acids, oleic acid, olive oil, skin care, type 2 diabetes

## Abstract

The research on health effects of olive oil and oleic acid remains variable. This umbrella review aimed to synthesize evidence from systematic reviews (with or without meta‐analyses) regarding the health outcomes of oleic acid and olive oil, clarify the validity and strength of the evidence, and finally help to make dietary recommendations for different people. We conducted a comprehensive search of the Cochrane, PubMed, Web of Science, Medline, and Embase databases. These studies were assessed using AMSTAR 2 and GRADE tools. 19 out of 741 eligible studies were included, and the majority were prospective studies. Topical application of olive oil is suggested to prevent pressure ulcers and acute radiation dermatitis; only a few adverse reactions (e.g., occupational allergic contact dermatitis and lichen planus pigmentosus) have been reported. Consumption of olive oil reduced the risk of Type 2 diabetes and cardiovascular disease by 22% and 18%, respectively. Especially, olive oil improved glycemic control in reducing fasting blood glucose (MD: −0.44, 95% CI: −0.66, −0.22), HbA1c (MD = −0.27, 95% CI: −0.37, −0.17), insulin (SMD = −0.28, 95% CI: −0.51, −0.05), and homeostatic model assessment for insulin resistance (HOMA‐IR) (SMD = −0.19, 95% CI: −0.35, −0.03). Every 25 g/day OO intake was associated with an 11% lower relative risk of all‐cause mortality (95% CI: 0.85, 0.93). Additionally, each 10 g/day olive oil intake increased 0.22 mg/dL HDL and improved endothelial function. However, the quality of 77% of the evidence was low to moderate, as assessed by GRADE. The methodological quality of 69% of the studies was low and critically low. Olive oil is proven to be a healthy dietary option for Type 2 diabetes and cardiovascular disease populations, a superior topical application choice for the immobilized. With the major evidence not being high, further standardized research is needed to explore the optimal dosage and the effects of oleic acid in olive oil.

## Graphical abstract

We conducted a comprehensive search of olive oil and oleic acid in 5 databases, and 19 out of 741 were selected. These studies were independently assessed and summarized by using AMSTAR 2 and GRADE tools. Olive oil is beneficial for pressure ulcers, acute radiation dermatitis, type 2 diabetes, and cardiovascular diseases.

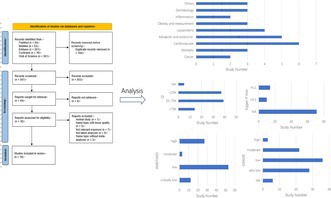

**Abbreviations**
AMSTAR: assessment of multiple systematic reviews
BMI: body mass index
CHD: coronary heart disease
CRP: C‐reactive protein
CVD: cardiovascular Disease
EVOO: extra virgin olive oil
FBS: fasting blood sugar
GRADE: Grading of Recommendations Assessment, Development, and Evaluation
HDL: high‐density lipoprotein
IR: insulin resistance
LDL: low‐density lipoprotein
MedDiet: mediterranean diet
MetS: metabolic syndrome
MUFA: monounsaturated fatty acids
NALFD: non‐alcoholic fatty liver disease
NO: nitric oxide
OA: oleic acid
OO: olive oil
PU: pressure ulcer
PUFA: polyunsaturated fatty acid
RCT: randomized control trials
SFA: saturated fatty acids
T2D: type 2 diabetes
TC: total cholesterol
TG: triglycerides
VOO: virgin olive oil
WC: waist circumference
WHR: waist‐to‐hip ratio

## 1 Introduction

Olive oil (OO) is a major fat source in the traditional Mediterranean diet (MedDiet), which is recognized a healthy eating pattern for being low in saturated fat and high in vegetable oils (Davis et al. 2015). Physically extracted from various olive cultivars, it holds significant cultural and economic importance, especially in Mediterranean countries, which are the biggest OO producers as well as consumers (Knowlton 2013; Uylaşer and Yildiz 2014). OO primarily consists of triglycerides (97%–99%), with unsaturated fatty acids accounting for approximately 85% of its fatty acid profile. The predominant monounsaturated fatty acid (MUFA) is oleic acid (OA; C18:1 n‐9), while saturated fatty acids (SFAs) such as palmitic and stearic acids make up about 14% (Jimenez‐Lopez et al. 2020). The remaining proportion includes minor bioactive compounds, such as polyphenols (e.g., squalene, oleuropein and its metabolites hydroxytyrosol and tyrosol), phytosterols, and carotenoids, which contribute to OO's unique bitter and acrid taste, and many of its health properties (Knowlton 2013; Jimenez‐Lopez et al. 2020; Chrysant and Chrysant 2022; Vitaglione et al. 2015).

Six OOs types are as followed: EVOO, virgin olive oil (VOO), refined OO, OO, refined pomace oil, and olive pomace oil. EVOO is considered to be of the highest quality, because it is solely extracted by physical means without chemical processes, which preserves more (poly)phenolic compounds (Jimenez‐Lopez et al. 2020). Polyphenols from EVOO underpin many properties, such as antihypertensive, hypolipidemic, antioxidative, anti‐inflammatory, platelet antiaggregating, antidiabetic. They also improve endothelial function, modulate intestine microbiota and decrease autophagy (Chrysant and Chrysant 2022; Perez‐Jimenez et al. 2005; López‐Miranda et al. 2010; Gaforio et al. 2019; Visioli et al. 2002). Meanwhile, the high MUFA content—predominantly oleic acid, lowers total cholesterol, low‐density lipoprotein (LDL), and the ratio of total cholesterol (TC)/high‐density lipoprotein (HDL), while also inhibiting platelet aggregation (Yubero‐Serrano et al. 2019). Notably, polyphenols act synergistically with MUFAs to delay postprandial lipemia, thereby reducing cardiovascular risk (Yubero‐Serrano et al. 2019). Additionally, the oleic acid itself may potentially inhibit cancer risk by suppressing oncogene HER2 (Colomer and Menéndez 2006). The presence of oleic acid, squalene and antioxidant substances further supports the dermatological benefits of OO. They exert anti‐inflammatory, antineoplastic, and antiaging function, which may explain why it helps the healing of pressure ulcers and recovery of burns in animal experiments (Lin et al. 2018; Viola and Viola 2009).

While harmful doses of OO intake have not been established, recommended daily dosages from the European Food Safety Authority (EFSA) and the US Food and Drug Administration (FDA) include more than 2 spoons (23 g) with established (poly)phenol content per day for at least 3 weeks (EFSA) and exactly 2 tablespoons (23 g) daily for heart protection (FDA), respectively (Tomé‐Carneiro et al. 2020). In the PREDIMED study, the recommendation of the EVOO intake was more than 50 g per day for the primary prevention of cardiovascular disease (Estruch et al. 2018a).

Given the diverse composition of OO, it is challenging to ascertain which specific compound is solely responsible for its observed health effects. While many studies focus on the minor compounds of OO, oleic acid, comprising approximately 80% of OO, also demonstrates significant health benefits (Lopez et al. 2014). Up to now, hundreds of randomized control trials (RCTs), case–controls, cohorts, and systematic reviews of them about relations between OO and health or diseases have been done, but the outcomes are controversial (Wang et al. 2022; Dehghani et al. 2021; Morvaridzadeh et al. 2023; Martínez‐González et al. 2022; Fernandes et al. 2020; Schwingshackl et al. 2017; Markellos et al. 2022). Therefore, to reconcile existing evidence and evaluate its robustness, we conducted this umbrella review of systematic reviews and meta‐analyses focusing on OO and oleic acid. Our aims are to provide a higher level of evidence, assess the quality of that evidence, and identify current research gaps.

## 2 Methods

### 2.1 Umbrella Review Methods

An umbrella review should begin with a clearly defined topic and a pre‐established, transparent methodology, which includes rigorous assessment of the quality and credibility of existing literature to support evidence‐based clinical decision‐making (Fusar‐Poli and Radua 2018; Aromataris et al. 2015; Ao et al. 2022). Our research was registered in the International Prospective Register of Systematic Reviews (PROSPERO CRD420251146945).

### 2.2 Search Strategy

We searched and analyzed the systematic reviews (with or without meta‐analyses) about OO or oleic acid and its effects on health. Databases including Cochrane, PubMed, Web of Science, Medline, and Embase were searched until November 22, 2023. The key search strategy was as follows: (olive oil or oleic acid) And (systematic review and meta‐analys*), detail search strategy was provided in the Supporting Information. No language restriction. We also searched the references of the eligible studies in case we missed out on any. Disagreements were resolved through consensus or discussion with the third researcher.

### 2.3 Inclusion Criteria and Exclusion Criteria

The inclusion criteria were as follows: (1) the article was a meta‐analysis with/without systematic review of interventional and/or observational studies; (2) evaluated the association of OO or its oleic acid component and human health outcomes. The exclusion criteria were: (1) reviews unrelated to OO; (2) themed on MUFA from unspecified source; (3) studies focus on (poly)phenols; (4) themes of MedDiet, dietary patterns where OO was not monitored as an independent intervention; (5) the full text was not available; (6) animal experiments and in vitro experiments; (7) narrative reviews and mechanism studies. All inclusion criteria must be met for inclusion, while only any single exclusion criterion satisfied was sufficient for exclusion. If more than one systematic review themed on the same outcome, we included the latest one with largest participants and excluded the same topic but without meta‐analysis.

### 2.4 Data Extraction

Two reviewers retrieved data independently from included articles as follows: health outcomes, type of OO or OA and comparison, the first author's name, publication year, number of cases and participants, study design, number of studies of different types, OO or OA dosage and duration, the estimate synthesized effect (OR, HR, MD, ME, RD, WMD), 95% confidence intervals, heterogeneity, publication bias, the type of effect model (random or fixed), and dose–response analyses. If any discrepancies that were unable to be solved by consensus would be resolved by a third author, who made the final decision.

### 2.5 Evidence Evaluation and Grading

The updated AMSTAR 2 (assessment of multiple systematic reviews) is to assess the methodological quality of random or nonrandom studies. AMSTAR 2 has 16 items and the evaluation options are “Yes”, “Partial yes,” and “No”. The critical items include: 2, 4, 7, 9, 11, 13, and 15. The review can be rated “high,” “moderate,” “low”, and “critically low” based on no or one noncritical weakness, more than one noncritical weakness, one critical flaw with or without noncritical weaknesses, more than one critical flaw with or without noncritical weaknesses (Shea et al. 2017). Reviewers referred to the Grading of Recommendations Assessment, Development, and Evaluation (GRADE) system to classify the strength of the evidence as “very low,” “low,” “moderate,” or “high” (Guyatt et al. 2011).

### 2.6 Data Analysis

The estimated summary effect with 95% CI and dose–response was extracted from every eligible article. The heterogeneity was calculated by the *I* ^2^ metric and Cochran's *Q* test. The type of effect model depends on the *I* ^2^ metric which means high heterogeneity and application of a random model if it is over 50. The publication bias was assessed by Egger regression and the symmetry of the Funnel plot. The *p*‐value of Cochran's *Q* test and Egger test less than 0.1 was deduced as statistically significant. The significance threshold of *p* < 0.05 was applied in other tests.

## 3 Results

### 3.1 Characteristics of the Study

**FIGURE 1:**
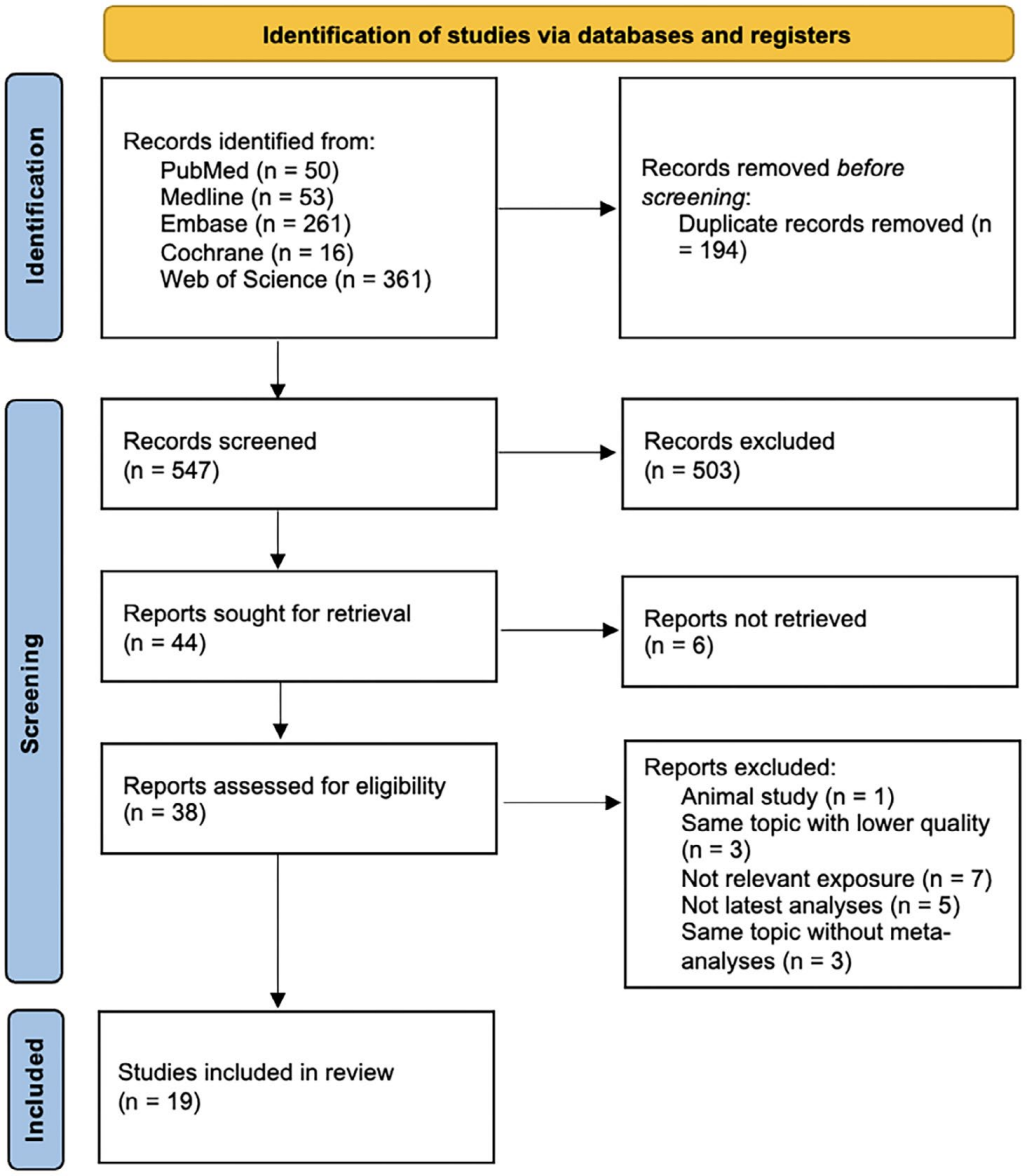
PRISMA flow diagram.

**FIGURE 2:**
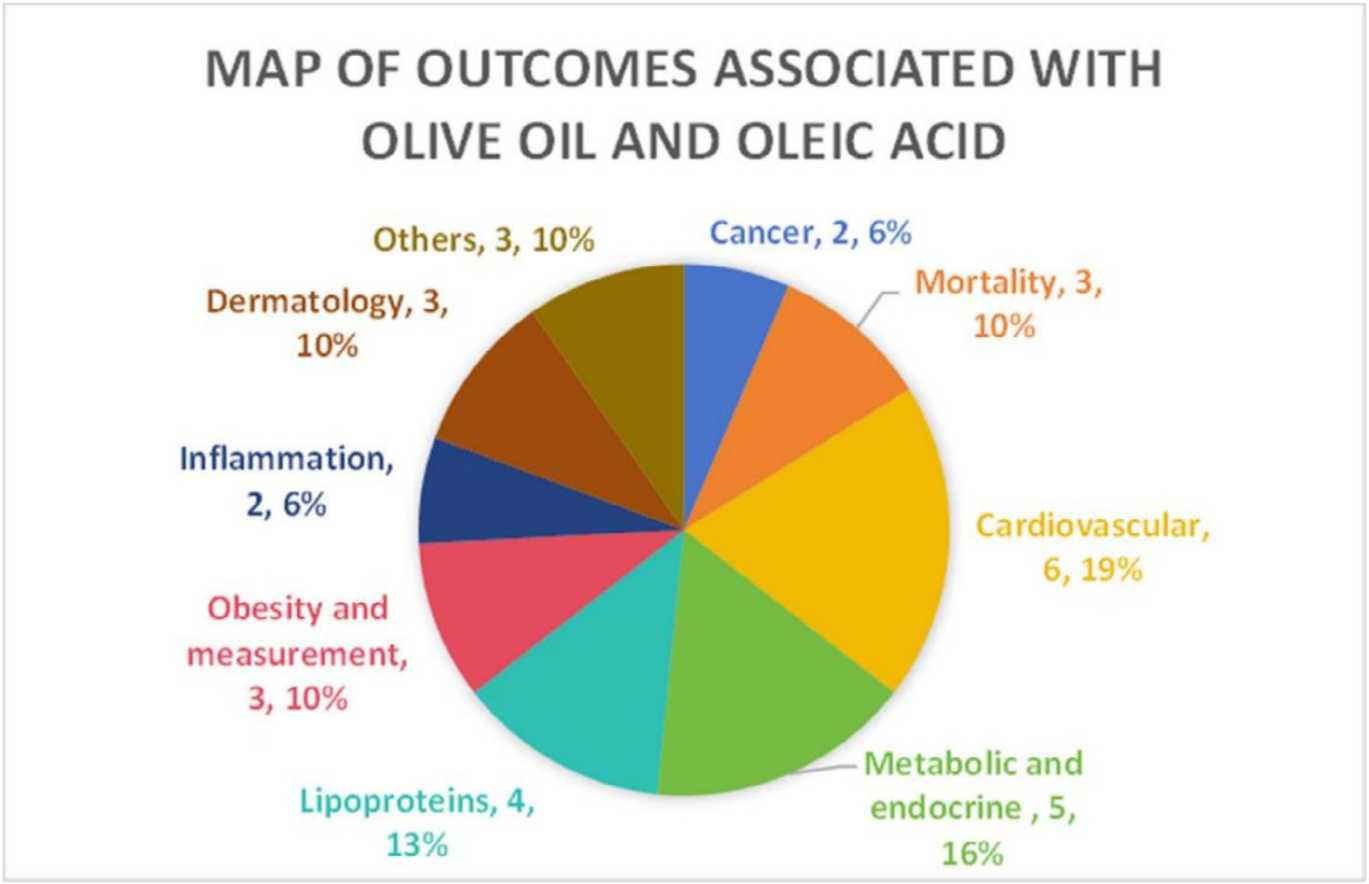
Health outcomes of olive oil and oleic acid.

Figure 1 shows the flow diagram of the article selection process. A total of 741 articles were retrieved after our systematic search. We imported the retrieved literature into Endnotes, conducted automated screening for duplicates, and then performed manual filtering. 194 duplicates were removed. After screening for title and abstract, 503 articles were excluded and 6 reports were not retrieved. Referring to the inclusion and exclusion criteria mentioned before, finally 19 articles were selected. Figure 2 showed more than 8 outcomes related to OO or OA use. Most studies are randomized controlled trials and cohort studies (Tables 1, 2, 3, 4, 5, 6, 7, 8, 9). Details of the characteristics of the studies are in Tables S1–S9.

**TABLE 1:** Endocrine and metabolic outcomes.

Outcome	Category	Study	No. of cases/participants	MA metric	Estimates	95% CI	No. of studies in MA	Cohort	Case control	RCT	Effects model	*I* ^2^ (%)	Egger test, *p*	AMSTAR2	GRADE
Risk of T2D	OO	Schwingshackl et al. (2017)	19,081/185,671	RR	0.84	0.77, 0.92	5	4	0	1	Random	22	NA	Critically Low	Low
HbA1c (%)	OO	Schwingshackl et al. (2017)	1428	MD	−0.27	−0.37, −0.17	22	0	0	22	Random	0	NA	Low	Moderate
FBS (mmol/L)	OO	Schwingshackl et al. (2017)	1724	MD	−0.44	−0.66, −0.22	25	0	0	25	Random	26	NA	Low	Moderate
FBS	EVOO	Morvaridzadeh et al. (2023)	909	SMD	0.05	−0.08,0.18	16	0	0	16	Random	0.00	NA	Low	Moderate
Insulin	EVOO	Morvaridzadeh et al. (2023)	626	SMD	−0.28	−0.51, −0.05	10	0	0	10	Random	48.57	NA	Low	High
HOMA‐IR level	EVOO	Morvaridzadeh et al. (2023)	583	SMD	−0.19	−0.35, −0.03	9	0	0	9	Random	0.00	NA	Low	High
Total T2D (incidence and mortality combined)	OO	Neuenschwander et al. (2023)	13,164/666,207	SHR	0.94	0.91,0.98	3	3	0	0	Random	12.10	0.29	Low	Low
T2D (incidence or mortality)	OO	Martínez‐González et al. (2022)	13,389/680,239	RR	0.78	0.69,0.87	4	3	0	1	Random	0	0.51	Low	Moderate
117 factors of MetS	OA	Pastor et al. (2021)	NA	SMD	0.03	−0.01,0.07	NA	0	0	NA	Random	0.00	NA	Critically low	High
Glycemic profile	OA	Pastor et al. (2021)	951	SMD	0.04	−0.10,0.18	6	0	0	6	Random	12.00	NA	Critically low	Low
246 factors of MetS	OO	Pastor et al. (2021)	NA	SMD	−0.01	−0.05,0.03	NA	0	0	NA	Random	55.00	NA	Critically low	Low
Glycemic profile	OO	Pastor et al. (2021)	2772	SMD	−0.00	−0.12,0.11	12	0	0	12	Random	40.00	NA	Critically low	Low

Abbreviations: MA, meta‐analysis; NA, not available; OA, oleic acid; RR, ratio risk; SHR, summary hazard ratio; SMD, standardized mean difference.

### 3.2 Endocrine and Metabolic Outcomes

#### 3.2.1 Low‐Quality Evidence

We observed that OO consumption decreased the combined risk of incidence and mortality of Type 2 diabetes (T2D) by 6% (SHR = 0.94, 95% CI: 0.91, 0.98) (Neuenschwander et al. 2023) and risk of Type 2 diabetes (RR = 0.84, 95% CI: 0.77, 0.92, *p* < 0.01) (Schwingshackl et al. 2017). OA consumption was not correlated with glycemic profile (SMD = 0.04, 95% CI: −0.10, 0.18, *p* = 0.59) (Pastor et al. 2021). Similarly, the assessment of OO showed no effect on 246 factors related to Metabolic syndrome (MetS) (SMD = −0.02, 95% CI: −0.10, 0.05) and glycemic profile (SMD = −0.00, 95% CI: −0.12, 0.11, *p* = 0.95) (Pastor et al. 2021). Pastor et al. included studies which compared OO to other oils which have already demonstrated their beneficial effects on MetS, which may cause insignificant results (Table 1).

#### 3.2.2 Moderate‐Quality Evidence

OO oral use lowered the 22% risk of T2D incidence or mortality with every additional 25 g/day consumption in OO (RR = 0.78, 95% CI: 0.69, 0.87) (Martínez‐González et al. 2022). The use of OO caused a pronounced reduction in HbA1c (MD: −0.27, 95% CI: −0.37, −0.17, *p* < 0.01) and fasting glucose value in T2D (MD = −0.44, 95% CI: −0.66, −0.22, *p* < 0.01) (Schwingshackl et al. 2017). But EVOO had no influence on FBS in a population with a low proportion of T2D (SMD = 0.05, 95% CI: −0.08, 0.18) (Morvaridzadeh et al. 2023).

#### 3.2.3 High‐Quality Evidence

The global effect of OA on 117 factors related to Metabolic syndrome (MetS) (SMD = 0.03, 95% CI: −0.01, 0.07) was insignificant (Pastor et al. 2021). This is related to the high number of studies showing low precision of individual results by including the null value within their confidence interval. A higher dose (at least above 25 g/day) intake of EVOO with an average duration of above 8 weeks significantly decreased insulin (SMD = −0.28, 95% CI: −0.51, −0.05) and homeostatic model assessment for insulin resistance (HOMA‐IR) (SMD = −0.19, 95% CI: −0.35, −0.03) (Morvaridzadeh et al. 2023).

### 3.3 Anthropometric Indices

#### 3.3.1 Very‐Low‐Quality Evidence

There was no difference in the mean body fat (in kilograms) between the OO intervention and comparator groups (MD = −0.24 kg, 95% CI: −0.85, 0.37). Mean hip circumference was also higher with OO consumption but without a significant difference (MD = 1.31, 95% CI: −0.24, 2.86) (Santos et al. 2023; Table 2).

**TABLE 2:** Anthropometric indices outcomes.

Outcome	Category	Study	No. of cases/participants	MA metric	Estimates	95% CI	No. of studies in MA	RCT	Effects model	*I* ^2^ (%)	Egger test, *p*	AMSTAR 2	GRADE
BMI (kg/m^2^)	OO as food/culinary ingredient or in capsules	Santos et al. (2023)	2842	MD	−0.05	−0.23, 0.13	41	41	Fixed	0	NA	High	Low
WC (cm)	OO as food/culinary ingredient or in capsules	Santos et al. (2023)	2533	MD	0.28	−0.22, 0.78	33	33	Fixed	15	NA	High	Low
WC (cm)	OO in capsules	Santos et al. (2023)	NA	MD	1.74	0.86, 2.62	12	12	Fixed	31	NA	Low	Na
WC (cm)	OO as a culinary ingredient/food	Santos et al. (2023)	NA	MD	−0.42	−1.03, 0.19	21	21	Fixed	0	NA	Low	Na
Hip circumference (cm)	OO	Santos et al. (2023)	470	MD	1.31	−0.24, 2.86	9	9	Fixed	0	NA	High	Very low
WHR	OO	Santos et al. (2023)	634	MD	0	−0.01, 0.02	12	12	Fixed	0	NA	High	Moderate
Mean total body fat (kg)	OO	Santos et al. (2023)	1264	MD	−0.24	−0.85, 0.37	18	18	Fixed	33	NA	High	Very low
Mean total body fat (kg)	OO in capsules	Santos et al. (2023)	NA	MD	0.28	−0.27, 0.83	9	9	Fixed	38	NA	Low	Na
Mean total body fat (kg)	OO as a culinary ingredient/food	Santos et al. (2023)	NA	MD	−0.29	−0.72, 0.14	9	9	Fixed	19	NA	Low	Na
Mean total body fat/adipose mass (%)	OO intake	Santos et al. (2023)	1417	MD	0.02%	−0.57%, 0.61%	22	22	Fixed	0	NA	High	Moderate
Mean mussle mass/lean mass (kg)	OO intake	Santos et al. (2023)	1338	MD	−0.27	−0.58, 0.05	20	20	Fixed	11	NA	High	Low
Mean mussle mass/lean mass (kg)	OO in capsules	Santos et al. (2023)	NA	MD	−0.47	−0.89, −0.06	11	11	Fixed	0	NA	Low	Na
Mean mussle mass/lean mass (kg)	OO as a culinary ingredient/food	Santos et al. (2023)	NA	MD	0.04	−0.46, 0.53	9	9	Fixed	20	NA	Low	Na
Weight	EVOO	Morvaridzadeh et al. (2023)	930	SMD	−0.06	−0.19, 0.07	16	16	Random	0.00	NA	High	Moderate
BMI (kg/m^2^)	EVOO	Morvaridzadeh et al. (2023)	913	SMD	−0.04	−0.17, 0.09	17	17	Random	0.00	NA	High	Moderate
WC	EVOO	Morvaridzadeh et al. (2023)	728	SMD	−0.01	−0.16,0.13	13	13	Random	0.00	NA	High	Moderate
WHR	EVOO	Morvaridzadeh et al. (2023)	134	SMD	−0.08	−0.44,0.27	3	3	Random	0.00	NA	Low	Low
Body composition	OA	Pastor et al. (2021)	2155	SMD	−0.06	−0.17,0.05	9	9	Random	34.00	NA	low	Moderate

#### 3.3.2 Low‐Quality Evidence

The association between body mass index (BMI) and OO was not significant (MD = −0.05, 95% CI: −0.23, 0.13). We found a nonsignificant increase in waist circumference (WC) with the ingestion of OO (MD = 0.28, 95% CI: −0.22, 0.78). The assessment of lean mass (kg) was not different between the groups (MD = −0.27 kg, 95% CI: −0.58, 0.05) (Santos et al. 2023). We observed no change in waist‐to‐hip ratio (WHR) (*n* = 3, SMD = −0.08, 95% CI: −0.44, 0.27) (Morvaridzadeh et al. 2023).

#### 3.3.3 Moderate‐Quality Evidence

OO intake had no effect on WHR (MD = 0.00, 95% CI: −0.01, 0.02). When adiposity was evaluated as a percentage, there was no significant difference (MD = 0.02%, 95% CI: −0.57%, 0.61%) (Santos et al. 2023). The result displayed an insignificant reduction in weight (*n* = 16, SMD = −0.06, 95% CI: −0.19, 0.07), BMI (*n* = 17, SMD = −0.04, 95% CI: −0.17, 0.09), WC (*n* = 13, SMD = −0.01, 95% CI: −0.16, 0.13) (Morvaridzadeh et al. 2023). When assessing OA and body composition, the result is also not closely connected (SMD = −0.06, 95% CI: −0.17, 0.05) (Pastor et al. 2021).

### 3.4 Inflammation Markers

#### 3.4.1 Very‐Low‐Quality Evidence

We observed that OO consumption was negatively related to IL‐6 (MD = −0.29, 95% CI: −0.7, −0.02), sE‐Selection (MD = −3.16, 95% CI: −4.07, −2.25), sICAM‐1 and sVCAM‐1, positively linked to flow‐mediated dilatation (FMD%) (MD = 76%, 95% CI: 0.27, 1.24) and sP‐Selection (MD = 10.78, 95% CI: 4.01, 17.54), with no association between OO and adiponectin, TNF‐α (Schwingshackl et al. 2015). Moreover, EVOO displayed no association with the IL‐10 level (SMD = −0.06, 95% CI: −0.33, 0.21) (Morvaridzadeh et al. 2023; Table 3).

**TABLE 3:** Inflammation markers.

Outcomes	Category	Study	No. of cases/participants	MA metric	Estimates	95% CI	No. of studies in MA	RCT	Effects model	*I* ^2^ (%)	Egger test, *p*	AMSTAR 2	GRADE
lipoprotein a	EVOO	Morvaridzadeh et al. (2023)	231	SMD	−0.35	−1.02, 0.32	4	4	Random	83.96	NA	Low	Low
CRP	EVOO	Morvaridzadeh et al. (2023)	398	SMD	0.03	−0.20, 0.26	7	7	Random	21.96	NA	High	Moderate
IL‐6	EVOO	Morvaridzadeh et al. (2023)	286	SMD	0.07	−0.16, 0.30	5	5	Random	0.00	NA	Low	Low
IL‐10	EVOO	Morvaridzadeh et al. (2023)	207	SMD	−0.06	−0.33, 0.21	3	3	Random	0.00	NA	Low	Very low
TNF‐α	EVOO	Morvaridzadeh et al. (2023)	120	SMD	0.03	−0.32, 0.38	3	3	Random	0.00	NA	Low	Low
CRP	OO	Schwingshackl et al. (2015)	1272	MD	−0.64	−0.96, −0.31	15	15	Random	66.00	NA	low	Low
IL‐6	OO	Schwingshackl et al. (2015)	857	MD	−0.29	−0.7, −0.02	7	7	Random	62.00	NA	Critically low	Very low
FMD%	OO	Schwingshackl et al. (2015)	851	MD	76%	0.27, 1.24	8	8	Random	26.00	NA	Critically low	Very low
Adiponectin	OO	Schwingshackl et al. (2015)	313	MD	0.44	−0.20, 1.09	6	6	Random	56.00	NA	Critically low	Very low
TNF‐α	OO	Schwingshackl et al. (2015)	303	MD	0.02	−0.02, 0.07	5	5	Random	95.00	NA	Critically low	Very low
sE‐Selectin	OO	Schwingshackl et al. (2015)	187	MD	−3.16	−4.07, −2.25	2	2	Random	0.00	NA	Critically low	Very low
sP‐Selectin	OO	Schwingshackl et al. 2015	358	MD	10.78	4.01, 17.54	4	4	Random	41.00	NA	Critically low	Very low
sICAM‐1	OO	Schwingshackl et al. (2015)	724	MD	−0.02	−0.04, 0.00	7	7	Random	84.00	NA	Critically low	Very low
sVCAM‐1	OO	Schwingshackl et al. (2015)	524	MD	−0.02	−0.05, 0.01	8	8	Random	37.00	NA	Critically low	Very low

Abbreviations: FMD%, flow‐mediated dilatation; SD, standard deviation; sICAM‐1, soluble intracellular adhesion molecule‐1; sVCAM‐1, soluble vascular cellular adhesion molecule‐1; WMD, weighted mean differences.

#### 3.4.2 Low‐Quality Evidence

Regarding inflammatory cytokines, IL‐6 (SMD = 0.07, 95% CI: −0.16, 0.30), lipoprotein a, and TNF‐α (SMD = 0.03, 95% CI: −0.32, 0.38) were irrelevant to EVOO consumption (Morvaridzadeh et al. 2023). Also, OO had no effect on lipoprotein A1 and lipoprotein B (Ghobadi et al. 2019). Another study showed notable results of OO and CRP (MD = −0.64, 95% CI: −0.96, −0.31) (Schwingshackl et al. 2015).

#### 3.4.3 Moderate‐Quality Evidence

The EVOO consumption had no association with the CRP level (SMD = 0.03, 95% CI: −0.20, 0.26) (Morvaridzadeh et al. 2023). The synthetic results of CRP and IL‐6 from the included meta‐analyses varied for a couple of reasons: the number of studies ranging from 5 to 15, the differences in study design, and the diversity in the included population. *I* ^2^ of Lukas Schwingshackl's results (MD) is over 60%, while that of Mojgan Morvaridzadeh's (SMD) is below 25%, which makes the significance of the MD results unreliable.

### 3.5 Cardiovascular Diseases

#### 3.5.1 Very‐Low‐Quality Evidence

MUFA from multiple sources reduced 9% and 17% risk of combined cardiovascular events (RR = 0.91, 95% CI: 0.86, 0.96) and stroke (RR = 0.83, 95% CI: 0.71, 0.97), respectively, and also tended to reduce coronary heart disease (CHD) (RR = 0.96, 95% CI: 0.90, 1.01) (Schwingshackl and Hoffmann 2014; Table 4).

**TABLE 4:** Cardiovascular diseases.

Outcomes	Category	Study	No. of cases/participants	MA metric	Estimates	95% CI	No. of studies in MA	Cohort	Case control	RCT	Effects model	*I* ^2^ (%)	Egger test, *p*	AM‐STAR 2	GRADE
SBP	OO	Zamora‐Zamora et al. (2018)	NA/6651	MSE	−0.11 mmHg	−0.68, 0.46	13	0	0	13	Fixed	85.10	0.67	Low	Low
DBP	OO	Zamora‐Zamora et al. (2018)	NA/6651	MSE	−0.73 mmHg	−1.07, −0.40	15	0	0	15	Fixed	84.50	0.23	Low	Low
SBP	EVOO	Mojgan Morvaridzadeh et al. (2023)	525	SMD	−0.04	−0.33, 0.25	9	0	0	9	Random	63.02	NA	Low	Low
DBP	EVOO	Morvaridzadeh et al. (2023)	525	SMD	−0.11	−0.38, 0.16	9	0	0	9	Random	56.26	NA	High	Low
Total CVD (incidence of CVD, CHD, MI, and CVD mortality)	OO	Neuenschwander et al. (2023)	54,578/614,098	SHR	0.96	0.94, 0.98	3	3	0	0	Random	0	0.65	Low	Moderate
CVD mortality	OO	Neuenschwander et al. (2023)	47,568/613,503	SHR	0.96	0.94, 0.98	3	3	0	0	Random	0	1.00	Low	Moderate
CVD	OO	Martínez‐González et al. (2022)	49,223/806,203	RR	0.84	0.76, 0.94	13	12	0	1	Random	67.90	< 0.001	High	Moderate
CVD (CHD and stroke)	OO	Martinez‐Gonzalez et al. (2014)	NA/140,133	RR	0.82	0.70, 0.96	9	5	3	1	Random	77.00	0.06	High	Moderate
CHD	OO	Martinez‐Gonzalez et al. (2014)	NA/101,460	RR	0.87	0.72, 1.05	8	4	3	1	Random	77.00	0.06	High	Moderate
Stroke	OO	Martinez‐Gonzalez et al. (2014)	NA/38,673	RR	0.76	0.67, 0.86	3	0	2	1	Random	0.00	0.11	High	Moderate
Cardiovascular mortality	MUFA	Schwingshackl and Hoffmann (2014)	388,334	RR	0.88	0.80, 0.96	14	14	0	0	Random	50	0.12	Low	Low
Combined cardiovascular events	MUFA	Schwingshackl and Hoffmann (2014)	813,102	RR	0.91	0.86, 0.96	30	30	0	0	Random	58	0.01	Low	Very low
CHD	MUFA	Schwingshackl and Hoffmann (2014)	NA	RR	0.96	0.90, 1.01	15	15	0	0	Random	41	0.28	Low	Very low
Stroke	MUFA	Schwingshackl and Hoffmann (2014)	NA	RR	0.83	0.71, 0.97	11	11	0	0	Random	70	0.28	Low	Very low

Abbreviations: BP, blood pressure; CHD, coronary heart disease; CVD, cardiovascular disease; DBP, diastolic blood pressure; MSE, mean standard error; RR, relative risk; SBP, systolic blood pressure; SHR, summary hazard ratio.

#### 3.5.2 Low‐Quality Evidence

OO (liquid and capsules), mostly owing to EVOO, decreased DBP than other fats significantly (*n* = 15, MSE = −0.73 mmHg, 95% CI: −1.07, −0.40), whereas it was not connected with SBP (MSE = −0.11 mmHg, 95% CI: −0.68, 0.46) (Zamora‐Zamora et al. 2018). However, pooled analysis indicated the insignificant impacts of EVOO on DBP (SMD = −0.11, 95% CI: −0.38, 0.16) and SBP (SMD = −0.04, 95% CI: −0.33, 0.25) (Morvaridzadeh et al. 2023). Because the former's intervention measures use OO as the primary fat source in certain dietary patterns (Mediterranean diet, low‐fat diet), while the latter only focuses on OO consumption. The former lasts at least 3 months, longer than the latter's minimum 2 weeks.

#### 3.5.3 Moderate‐Quality Evidence

OO was inversely associated with stroke (RR = 0.76, 95% CI: 0.67, 0.86) and CVD (stroke and CHD combined) (RR = 0.82, 95% CI: 0.70, 0.96) but not with CHD statistically (RR = 0.87, 95% CI: 0.72, 1.05) (Martinez‐Gonzalez et al. 2014). OO decreased the 16% risk of CVD incidence (RR = 0.84, 95% CI: 0.76, 0.94) for every additional 25 g/day of OO consumption (Martínez‐González et al. 2022). OO intake decreased 4% risk of incidence of total CVD (SHR = 0.96, 95% CI: 0.94, 0.98) for substitution of butter with OO (5 g/day) (Neuenschwander et al. 2023).

### 3.6 Blood Lipids

#### 3.6.1 Very‐Low‐Quality Evidence

Each 10 g/day increment in OO consumption decreased LDL by 0.04 mg/dL (MD = −0.04, 95% CI: −1.01, 0.94); dose‐dependent effects of OO on levels of serum LDL indicated no change in LDL with the increase of OO intake (P nonlinearity = 0.61, P dose–response = 0.61) (Jabbarzadeh‐Ganjeh et al. 2023). With respect to oral fat tolerance tests on postprandial triglycerides, no difference between MUFA and SFA meals was found over 4 h (SMD = 0.70, 95% CI: −0.07, 1.41, *p* = 0.08), but there is a tendency for lower triglycerides (TG) to mainly OO‐sourced MUFA meals over 8 h (SMD = −0.89, 95% CI: −1.82, 0.04, *p* = 0.06) (Monfort‐Pires et al. 2016; Table 5).

**TABLE 5:** Blood lipids.

Outcome	Category	Study	No. of cases/participants	MA metric	Estimates	95% CI	No. of studies in MA	RCT	Effects model	*I* ^2^	Egger test, *p*	AMSTAR 2	GRADE
TG	EVOO	Morvaridzadeh et al. (2023)	1395	SMD	−0.05	−0.17, 0.07	26	26	Random	19.07	NA	High	Moderate
TC	EVOO	Morvaridzadeh et al. (2023)	1483	SMD	0.07	−0.12, 0.26	27	27	Random	68.87	NA	High	Low
LDL	EVOO	Morvaridzadeh et al. (2023)	1524	SMD	0.05	−0.12, 0.22	28	28	Random	61.09	NA	High	Low
HDL	EVOO	Morvaridzadeh et al. (2023)	1524	SMD	0.13	0.03, 0.28	28	28	Random	54.42	NA	High	Low
VLDL	EVOO	Morvaridzadeh et al. (2023)	348	SMD	0.12	−0.14, 0.38	6	6	Random	30.31	NA	Low	Moderate
ApoA‐I	EVOO	Morvaridzadeh et al. (2023)	570	SMD	0.16	−0.17, 0.50	10	10	Random	73.76	NA	High	Low
ApoB	EVOO	Morvaridzadeh et al. (2023)	503	SMD	0.29	−0.06, 0.63	9	9	Random	72.12	NA	Low	Low
Lipid profile	OA	Rosario Pastor et al. (2021)	4532	SMD	0.06	−0.00, 0.12	16	16	Random	0.00	NA	Low	Low
Lipid profile	OO	Pastor et al. (2021)	NA	SMD	0.01	−0.05, 0.06	NA	NA	Random	32.00	NA	Low	Moderate
Postprandial triglycerides over 8 h	MUFA of OO	Monfort‐Pires et al. (2016)	157	SMD	−0.89	−1.82, 0.04	8	8	Random	92.00	NA	low	Very low
Postprandial triglycerides over 4 h	MUFA of OO (main) and high‐oleic sunflower oil	Monfort‐Pires et al. (2016)	359	SMD	0.7	−0.07, 1.47	18	18	Random	95.00	NA	Low	Very low
Postprandial triglycerides over 6 h	MUFA of OO (main) and high‐oleic sunflower oil	Monfort‐Pires et al. (2016)	249	SMD	−0.04	−0.84, 0.75	14	14	Random	93.00	NA	low	Low
TC	OO	Jabbarzadeh‐Ganjeh et al. (2023)	1574	MD	0.79	−0.08, 1.66	31	31	Random	57.00	0.17	High	Low
LDL	OO	Jabbarzadeh‐Ganjeh et al. (2023)	1547	MD	−0.04	−1.01, 0.94	31	31	Random	80.00	0.21	High	Very low
HDL	OO	Jabbarzadeh‐Ganjeh et al. (2023)	1685	MD	0.22	−0.01, 0.45	33	33	Random	38.00	0.36	High	Low
TG	OO	Jabbarzadeh‐Ganjeh et al. (2023)	1631	MD	0.39	−0.33, 1.11	32	32	Random	7.00	0.54	High	Low

Abbreviations: HDL‐c, high‐density cholesterol; LDL‐c, low‐density cholesterol; SD, standard deviation; SMD, standardized mean difference; TC, total cholesterol; TG, triglyceride.

#### 3.6.2 Low‐Quality Evidence

EVOO had no significant effect on the levels of TC, LDL, HDL, Apo A‐I, and Apo B when EVOO was compared with other kinds of oil analyzed as a whole (Morvaridzadeh et al. 2023). Each 10 g/day increment in OO intake slightly increased TC concentration (MD = 0.79, 95% CI: −0.08, 1.66); dose‐dependent effects (P nonlinearity = 0.41, P dose–response = 0.12) of OO on levels of TC showed that the TC levels increase with the OO consumption up to 30 g/day (MD 30 g/day = 2.76 mg/dL, 95% CI: 0.01, 5.51) and reach a plateau till 40 g/day. Also, each 10 g/day of OO increased HDL concentrations significantly (MD = 0.22 mg/dL, 95% CI: −0.01, 0.45); dose‐dependent effects of OO showed a small increase in HDL‐cholesterol concentration (P nonlinearity = 0.22, P dose–response = 0.05). However, there is no statistically significant change in triglyceride (TAG) concentration for each 10 g/day of OO (MD = 0.39 mg/dL, 95% CI: −0.33, 1.11) and a nonsignificant increase in TAG with the increase of OO consumption in the dose–response test (P nonlinearity = 0.33, P dose–response = 0.32) (Jabbarzadeh‐Ganjeh et al. 2023). Correlation between OA and lipid profile is insignificant (SMD = 0.06, 95% CI: −0.00, 0.12, *p* = 0.05) (Pastor et al. 2021). MUFA had no effects on oral fat tolerance tests on postprandial triglycerides over 6 h (SMD = −0.04, 95% CI: −0.84, 0.75, *p* = 0.91) (Monfort‐Pires et al. 2016). EVOO increased the level of HDL‐c compared with other oils (SMD = 0.13, 95% CI: 0.03, 0.28) (Morvaridzadeh et al. 2023), while the HDL‐c outcome is different in Jabbarzadeh‐Ganjeh's study (MD = 0.22, 95% CI: −0.01, 0.45) (Jabbarzadeh‐Ganjeh et al. 2023). The measurement results and duration of the two are different, and the intervention measures of the latter include not only EVOO but also VOO and ROO.

#### 3.6.3 Moderate‐Quality Evidence

A null effect of OO (SMD = 0.01, 95% CI: −0.05, 0.06, *p* = 0.81) was observed on lipid profile (Pastor et al. 2021). There is no difference between the EVOO group and other kinds of groups in TG and VLDL levels (Morvaridzadeh et al. 2023).

### 3.7 All‐Cause Mortality

#### 3.7.1 Very‐Low‐Quality Evidence

The risk of all‐cause mortality was significantly reduced when correlated with MUFA from multiple sources (RR = 0.89, 95% CI: 0.83, 0.96) (Schwingshackl and Hoffmann 2014; Table 6).

**TABLE 6:** Mortality.

Outcome	Category	Study	No. of cases/participants	MA metric	Estimates	95% CI	No. of studies in MA	Cohort	RCT	Effects model	*I* ^2^	Egger test, *p*	AMSTAR 2	GRADE
All‐cause mortality	OO	Martínez‐González et al. (2022)	174,081/733,420	RR	0.89	0.85, 0.93	11	10	1	Random	65.20	0.00	High	Moderate
All‐cause mortality	All MUFA combined	Schwingshackl and Hoffmann (2014)	418,406	RR	0.89	0.83, 0.96	17	17	0	Random	64	0.04	Low	Very low

#### 3.7.2 Moderate‐Quality Evidence

OO showed a protective effect on all‐cause mortality (SHR = 0.94, 95% CI: 0.92, 0.97), and every 25 g/day OO intake was associated with an 11% lower relative risk of all‐cause mortality with potential heterogeneity (RR = 0.89, 95% CI: 0.85, 0.93) (Martínez‐González et al. 2022).

### 3.8 Cancer Outcomes

The combined outcomes of incidence and mortality of cancer were not associated with OO (RR = 0.94, 95% CI: 0.86, 1.03, GRADE: low), due to the inclusion of cohorts measuring cancer mortality, the long follow‐up (Martínez‐González et al. 2022). When cancer incidence is regarded as the sole outcome, OO's anticancer properties are unconvincing for low or very low certainty. Because a high proportion of case–control studies strengthens the bias. Compared to the lowest OO consumption, the highest OO demonstrated a pointed protective function on cancer carcinogenesis (RR = 0.69, 95% CI: 0.62, 0.77, GRADE: very low), especially for upper aerodigestive, overall gastrointestinal, urinary tract, and prostate cancer. Subgroup analyses of Mediterranean and non‐Mediterranean regions showed significant associations. However, high dose OO intake was not correlated to gastric and colorectal cancer prevention (Markellos et al. 2022). For breast cancer, OO consumption showed a protective function for women but with high heterogeneity (OR = 0.75, 95% CI: 0.56, 1.00, *I* ^2^ = 83%, GRADE: low). The dose–response analysis showed that breast cancer was not associated with a 14 g/day increase in OO intake (OR = 0.93, 95% CI: 0.83, 1.04) (Sealy et al. 2021; Table 7).

**TABLE 7:** Cancer outcomes.

Outcome	Category	Study	No. of cases/participants	MA metric	Estimates	95% CI	No. of studies in MA	Cohort	Case control	RCT	Effects model	*I* ^2^	Egger test, *p*	AMSTAR	AMSTAR 2	GRADE
Cancer (incidence or mortality)	OO	Martínez‐González et al. (2022)	58,892/1285,064	RR	0.94	0.86, 1.03	10	9	0	1	Random	55.80	0.02	10	High	Low
Risk for overall cancer	OO	Markellos et al. (2022)	29,830/975,434	RR	0.69	0.62, 0.77	45	8	37	0	Random	75.40	< 0.001	10	Low	Very low
Breast cancer risk	OO	Sealy et al. (2021)	7030/81,436	OR	0.75	0.56, 1.00	10	2	8	0	Random	83	NA	6.5	Moderate	Very low
Gastrointestinal cancer	OO	Markellos et al. (2022)	5902/545,313	RR	0.77	0.66, 0.89	15	13	2	0	Random	40.60	0.05	10	Low	Low
Upper aerodigestive cancers	OO	Markellos et al. (2022)	3535/9176	RR	0.74	0.60, 0.91	6	6	0	0	Random	32.70	NA	9	Low	low
Urinary tract cancers	OO	Markellos et al. (2022)	1856/4337	RR	0.46	0.29, 0.72	6	6	0	0	Random	72.90	NA	9	Low	Very low
Colorectal cancer	OO	Markellos et al. (2022)	3282/53,824	RR	0.9	0.79, 1.03	7	6	1	0	Random	0.00	NA	9	Low	Low
Esophageal cancer	OO	Markellos et al. (2022)	559/1797	RR	0.47	0.24, 0.93	3	3	0	0	Random	61.50	NA	9	Low	Very low
Gastric cancer	OO	Markellos et al. (2022)	1699/487,778	RR	0.75	0.53, 1.05	4	3	1	0	Random	62.00	NA	9	Low	Very low
Prostate cancer	OO	Markellos et al. (2022)	1388/2798	RR	0.61	0.40, 0.92	4	4	0	0	Random	30.00	NA	9	Low	Very low

### 3.9 Derma Application Outcomes

Typical application of OO reduces the incidence of pressure ulcers (PU) (RR = 0.56 95% CI: 0.39, 0.79, GRADE: moderate) and the local adverse effects are less likely to happen (RR = 0.39 95% CI: 0.06, 2.62, GRADE: low) (Hernandez‐Vasquez et al. 2022). With regard to skin care of preventing acute radiation dermatitis in cancer patients, OO significantly cut down the incidence of grade 2^+^ (RR = 0.66 95% CI: 0.51, 0.85, GRADE: low) and grade 3^+^ skin reactions (RR = 0.23 95% CI: 0.07, 0.76, GRADE: very low) respectively and has a tendency to reduce RTOG grade 1–2 incidence (RR = 0.98 95% CI: 0.56, 1.71, GRADE: very low) (Robijns et al. 2022; Table 8).

**TABLE 8:** Derma application outcomes.

Outcome	Category	Study	No. of cases/participants	MA metric	Estimates	95% CI	No. of studies in MA	RCT	Effects model	*I* ^2^	Egger test, *p*	AMSTAR	AMSTAR 2	GRADE
Incidence of RTOG grade 1–2	OO	Robijns et al. (2022)	130/156	RR	0.98	0.56, 1.71	2	2	Random	93	NA	8	Low	Very low
RTOG grade 2+	OO	Robijns et al. (2022)	92/156	RR	0.66	0.51, 0.85	2	2	Random	0.00	NA	8	Low	Low
RTOG grade 3+	OO	Robijns et al. (2022)	16/94	RR	0.23	0.07, 0.76	1	1	Random	NA	NA	8	Low	Very low
Incidence of Pressure Ulcers	EVOO	Hernandez‐Vasquez et al. (2022)	115/1344	RR	0.56	0.39, 0.79	4	4	Random	0.00	NA	10	Low	Moderate
Adverse Events	EVOO	Hernandez‐Vasquez et al. (2022)	4/1274	RR	0.39	0.06, 2.62	3	3	Random	0.00	NA	10	Low	Low

Abbreviation: RD, risk difference.

### 3.10 Other Outcomes

The studies enrolling participants older than 55 years old indicated OO intake had a favorable effect on cognitive performance (Fazlollahi et al. 2023). It is reported that OO intake ameliorated the severity of hepatic steatosis and decreased aspartate transaminase and alanine transaminase levels (Ma et al. 2023). A study showed OO consumption reduced adverse maternal and fetal outcomes (Cortez‐Ribeiro et al. 2023; Table 9).

**TABLE 9:** Other outcomes.

Outcome	Category	Study	No. of cases/participants	MA metric	Estimates	95% CI	No. of studies in SR	Cohort	Case control	RCT	Quasi‐experimental	Cross‐sectional	Effects model	*I* ^2^	Egger test, *p*	AMSTAR 2	GRADE
Maternal‐fetal outcomes (SGA and LGA newborns, GDM, preeclampsia, and cardiovascular risk)	EVOO as a supplement	Cortez‐Ribeiro et al. (2023)	44,644	NA	NA	NA	9	1	2	5	1	0	NA	NA	NA	Critically low	Very low
Cognitive fuction in the elder adults	Olive oil consumption	Fazlollahi et al. (2023)	14,040	NA	NA	NA	11	4	0	3	0	4	NA	NA	NA	Critically low	Very low
Hepatic steatosis	OO, EVOO, ROO, olive pomace oil	Ma et al. (2023)	344	NA	NA	NA	6	0	0	6	0	0	NA	NA	NA	Critically low	Low
Liver enzymes	OO, EVOO, ROO, olive pomace oil	Ma et al. (2023)	344	NA	NA	NA	5	0	0	5	0	0	NA	NA	NA	Critically low	Low

Abbreviations: GDM, gestational diabetes mellitus; LGA, large‐for‐gestational‐age; SGA, small‐for‐gestational‐age; SR, systematic review.

### 3.11 Adverse Events

Generally, the oral consumption and skin application of OO are safe. A rare report of a likely induction of Lichen Planus Pigmentosus in a woman (Haber et al. 2021). Some people may be specifically allergic to some substances of EVOO and get dermatitis (Ochi et al. 2021).

### 3.12 Heterogeneity

15% of the systematic reviews showed very high levels of heterogeneity (*I* ^2^ > 75%), 41% manifested moderate to high levels of heterogeneity (25% < *I* ^2^ < 75%), 39% presented low levels of heterogeneity (*I* ^2^ < 25%). The heterogeneity of the remaining 5% of the systematic reviews was not available.

### 3.13 Publication Bias

The majority of the studies (75%) did not report the publication bias, the Egger test of 15% of the studies showed *p* > 0.1 which meant a great confidence of no publication bias, only 11% of the studies showed a very likely possibility of publication bias (*p* < 0.1).

### 3.14 GRADE and AMSTAR 2 Classification

The quality of a few evidence (6%) cannot be acquired due to the lack of clear reporting of data for each outcome and assessment of bias risk. The quality of the major evidence (77%) was very low or low by GRADE. 23% of the outcomes were of moderate quality by GRADE. Only 3% of the evidence (insulin, HOMA‐IR level, 117 factors of MetS) was of high quality. For assessment of AMSTAR 2, 28% of the studies were rated as high, 3% were rated as moderate, 56% were rated as low, and 13% were rated as critically low. The details about GRADE and AMSTAR 2 of every outcome are shown in Tables 1, 2, 3, 4, 5, 6, 7, 8, 9 and Supporting Information S1.

## 4 Discussion

Generally, the associations between OO and health outcomes are evident in T2D, CVD, all‐cause mortality, and pressure ulcers.

In relation to endocrine and metabolic issues, EVOO demonstrates significant advantages in glycemic control. The ability of OO against oxidative stress plays a role in preventing the development of T2D (Schröder 2007). Although the capacity of ameliorating IR of OA is contentious, the underlying mechanisms of OA support the preventive effect on treating IR and T2D (Rehman et al. 2020). Studies found OA interferes with the interaction of β‐catenin with TCF7L2 and transfers β‐catenin to FoxO1 to inhibit IR (Jazurek‐Ciesiolka et al. 2019). Moreover, the intake of OO increases the fluidity of the membrane and the nonlamellar by elevating the ratio of MUFA or polyunsaturated fatty acid (PUFA) to SAF in T2D patients, which will reduce the binding of Gαi, Gαs protein, and PKC. The reduction of Gαs inhibits glucagon to regulate glycogenolysis and plasma glucose concentrations (Perona et al. 2007). That may elucidate OO consumption reduces FBS in T2D patients. Oleuropein and hydroxytyrosol from OO possess antioxidant capacity by activating the Nrf2/ARE pathway, which accounts for the low production of advanced glycosylated end‐products (AGEs) of T2D patients consuming OO. Consumption of hydroxytyrosol elevates the sensitivity of insulin and glucose tolerance. Phenolic acids and flavonoids of OO inhibit carbohydrate digestion and absorption (Alkhatib et al. 2018). All components of OO may take effect jointly to prevent T2D. The oils of the control group have been proven effective for MetS. Hence, the summary effect of OO on MetS is insignificant, which could suggest that OO was as good as the other strategies to manage MetS.

OO has no effects on anthropometric indices. Total energy intake, metabolic rate, and exercise energy expenditure greatly influence body composition. There is a tendency to reduce lean mass, especially when OO is supplemented in the capsule or consumed for a longer time; this may be related to lower consumption of total protein to avoid caloric excess (Santos et al. 2023). A study showed the intake of OO can decrease the BMI and weight (Gaforio et al. 2019). OO is rich in OA, which increases more fat oxidation and facilitates the daily energy expenditure subsequently when compared with linoleic and linolenic acids from other sources of oil (Jones et al. 2008). OA and linoleic acid are natural inhibitors of pancreatic lipase, which interferes with fat digestion and also reduces the intestinal absorption of triacylglycerols (Li et al. 2022).

Moreover, OO and MUFA consumption were very likely to reduce CVD and stroke according to the results, but the relation to CHD and BP was uncertain. While EVOO but not OO reduced BP in our umbrella review, studies conducted in spontaneous hypertensive rats revealed EVOO lowered the BP by antioxidative mechanisms for downregulation of NF‐κB and AP‐1, increased OA in cellular phospholipids regulated membrane lipid structure and decreased G protein‐coupled receptors (e.g., adrenoceptor α2A) (Estruch et al. 2018b; Terés et al. 2008). However, the BP protective function is evident in studies conducted in Mediterranean countries but not in Western‐like dietary pattern countries, where the intake of meat and SFA is high (Guasch‐Ferré et al. 2019). A PREDIMED study revealed MedDiet supplemented with a recommendation of EVOO at least 4 tablespoons per day primarily prevented CVD with 0.69 of HR (95% CI: 0.53, 0.91) (Estruch et al. 2018a). MUFAs were less susceptible to oxidation and improved lipid profiles, bioactive compounds had anti‐inflammatory and antioxidant properties, OO consumption enhanced endothelial function (Guasch‐Ferré et al. 2014). Atherosclerosis is considered to be an inflammatory disease in which monocytes and macrophages function and secrete proinflammatory cytokines, such as TNF‐α (Covas 2007). Since serum IL‐6 and CRP are predictors of atherosclerosis progression (Jialal et al. 2004), we found the effects of OO and OA on IL‐6 and CRP were uncertain in our umbrella review. However, in our study, the evidence for OO's protective effect against cardiovascular diseases is strong. Based on the mechanism of inflammation in cardiovascular disease development, we infer that OO intake has suppressing effects on inflammation. Both OA and polyphenols were well explained for CVD preventive mechanisms (Lu et al. 2024).

MUFA from multiple sources and OO manifested a reduction of CVD mortality and all‐cause mortality. In participants with high cardiovascular risk, the total OO consumption reduced 48% (HR = 0.56, 95% CI: 0.31, 1.02) of cardiovascular mortality, while EVOO made the most of it, but it was not associated with cancer. However, each 10 g/day increase of total OO and EVOO consumption was not significantly associated with all‐cause mortality with an average of 4.8 years follow‐up (Guasch‐Ferré et al. 2014). The follow‐up time is crucial for the results of all‐cause mortality. The studies included in our review were generally followed up for more than 4 years and even up to 30 years, which made the results differ.

Cancer outcomes were inconsistent for different types of cancer analyzed and mixed kinds of studies. However, a recent study with 18 years of follow‐up conducted in the Mediterranean region reported that two or more tablespoons per day had a 51% lower risk of cancer mortality (Torres‐Collado et al. 2022). In a synthesis of 19 observational studies in 2011, the highest OO consumption was associated with lower odds of any type of cancer development, predominantly breast cancer and cancer of the digestive system, which was irrespective of the country of origin (Psaltopoulou et al. 2011). OO may prevent colorectal cancer by altering secondary bile acid profiles in the colon, which could modulate polyamine metabolism in colonocytes (Stoneham et al. 2000). OO may reduce breast cancer risk, but the association is difficult to determine, whether it is due to OO specifically or to the overall Mediterranean diet (Sealy et al. 2021). Phenolic compounds inhibit tumorigenesis by targeting the HER2 (erbB‐2) oncogene signaling pathway in vitro experiments (Menendez et al. 2008). We previously introduced that oleic acid also targets HER2, so the relative contribution of MUFAs versus antioxidant compounds to the anticancer properties of OO is still not fully determined.

Compared to other vegetable oils, OO and EVOO did not exert a better reductive function of TG, TC, and LDL. However, EVOO significantly elevated HDL, and a dose–response effect was obvious. Other studies showed that the substitution of EVOO and its MUFA for SFA could reduce TC, LDL cholesterol, and TC/HDL cholesterol ratio (Yubero‐Serrano et al. 2019). The studies from our umbrella review included healthy and unhealthy groups, such as overweight, hypercholesterolemia, and nonalcoholic fatty liver disease (NALFD), which may have underlying effects on blood lipid profiles. Some comparisons of interventions may be specifically designed for benefiting blood lipid profiles or susceptible to lipid peroxidation, for example, n‐3 and n‐6 enriched oils or palm oil with high saturated fat (Pedersen et al. 2000; Hisham et al. 2020). Studies have shown that MUFA did not affect TC, while PUFA reduced TC, and SFA increased TC. However, MUFA consumption increased HDL levels more than PUFA and a carbohydrate‐rich diet (Covas 2007; Grundy 1989). Postprandial lipemia associated with oxidative changes was a risk factor for atherosclerosis development (Roche and Gibney 2000). BMI and sex are two important factors for the postprandial TG response (Sciarrillo et al. 2019). Also, studies found that an earlier and higher peak of TG after MUFA occurred than SFA in the first hours of the postprandial test (Monfort‐Pires et al. 2016). These reasons above may interpret no difference in postprandial TG between MUFA and SFA in the early hours but a tendency to lower TG of MUFA in 8 h.

We observed that the external application of OO contributes to the prevention of PU and acute radiation dermatitis. A pressure ulcer is caused by chronic oppression of the tissue leading to ischemia and necrosis or excess moisture (Mervis and Phillips 2019). Ischemia–reperfusion cycles, reactive oxygen species (ROS), and the inflammatory response are recognized as the characteristics of PU (Peirce et al. 2000). In a multicenter and double‐blind RCT conducted in the province of Cordoba, the incidence of PU was lower when OO was topically used compared with the hyperoxygenated fatty acid (HOFA) group, but the result was not statistically significant (PU incidence difference = −2.39%, 95% CI: −6.40%, 1.56%) (Díaz‐Valenzuela et al. 2019). Essential fatty acids like HOFAs improve skin hydration and elasticity and prevent skin breakdown in a poor nutritional status (Declair 1997). OO possesses OA as a major fatty acid, linoleic acids, phytosterols, and squalene and plays a role in skin care in a similar way (Díaz‐Valenzuela et al. 2019). Reduction of oxidative damage to proteins and lipids, infiltration of inflammatory cells, and promotion of PU wound healing were observed in a PU mice model treated with OO gavage compared with water (Donato‐Trancoso et al. 2016). Also, dietary OO supplementation stimulated NADPH oxidase, resulting in ROS generation, promoted nitric oxide (NO) synthesis and collagen deposition, reduced inflammation response by diminishing neutrophils and COX‐2 protein synthesis, and facilitated Nrf2 expression (Schanuel et al. 2019). Therefore, OO exerts skin protection function in external and internal pathways. OO is recommended as one of six interventions for the prevention and management of acute radiation dermatitis in MASCC clinical guidelines (Behroozian et al. 2023). The incidence of grade 1 dermatitis was lower in patients administered with additional OO and calcium hydroxide after receiving postmastectomy radiotherapy compared with the general skin care regimen group in an RCT. The mean Skindex‐16 score was better in the intervention group than in the control (*p* = 0.019) at the end of the trial (Chitapanarux et al. 2019). Combining with the results of our umbrella review, the protective effect of OO is more evident in the deterioration phase of dermatitis, meaning the profound skin benefits of OO prompt that early clinical intervention is necessary. Rare adverse events are reported in seldom individuals allergic to OO for skin use.

In our umbrella review, there is a protective function of OO intake and cognitive decline. A cohort study conducted in France with 6947 participants with 1–4 years follow‐up, those who consumed intensive or moderate amounts of OO performed better in cognitive tests including MMSE than nonusers. It is noteworthy that the association of intensive OO use and visual memory (adjusted OR = 0.83, 95% CI: 0.69–0.99) is significant, but verbal fluency is not obvious (OR = 0.85, 95% CI: 0.70–1.03) (Berr et al. 2009). Alzheimer's disease (AD) is characterized by the deposition of intraneuronal hyperphosphorylated tau protein and aggregation of extracellular misprocessed amyloid precursor protein (APP), which leads to neuronal loss and dementia (Panza et al. 2004). Evidence revealed oxidative stress might be directly involved in the development of toxic APP aggregation (Deschamps et al. 2001). Hypothetical explanations of MUFA and cognitive function are the relevant quota of tocopherol, polyphenols, and antioxidant compounds in OO, less intake of SFA, elevating tocopherol and polyphenols, cutting down low‐density lipoprotein cholesterol levels, affecting the structural integrity and fluidity of neuronal membranes, and thereby regulating neuronal transmission (Solfrizzi et al. 2005, 2010). Furthermore, in an elder population of Southern Italy with a typical Mediterranean diet, high MUFA intake showed a reverse association with age‐related cognitive decline (Solfrizzi et al. 1999). Studies revealed OO intake decreased fat in the liver and improved IR (Hussein et al. 2007). The decrease in liver enzyme levels was due to the minor active compounds of EVOO like phenols, oleocanthal, and hydroxytyrosol (Ma et al. 2023). Similarly, active compounds of EVOO benefited maternal‐fetal outcomes like gestational diabetes mellitus and preeclampsia (Cortez‐Ribeiro et al. 2023). However, the research themed on liver enzymes, hepatic steatosis, maternal‐fetal outcomes, and cognitive decline was not synthetically analyzed, so the bias is high.

## 5 Limitations

We identified 19 out of 741 articles after globally searching. With more than eight fields of associated health outcomes, the quality of the major evidence and the methodological quality are moderate, low, and very (critically) low, judged by GRADE and AMSTAR 2. These limitations substantially reduce the credibility of the conclusions across outcomes—including anthropometric indices, inflammation markers, blood lipids, cancer, and other outcomes—that have low credibility. Over half of the studies tended to have higher values of *I* ^2^ in this umbrella review, and significant heterogeneity also decreased the certainty of evidence. 75% of the studies did not report publication bias, causing a significant lack of transparency and potentially inflating effects in the original meta‐analyses. As we excluded duplicate studies during article screening, this may affect our interpretation of the pooled results in the umbrella review.

## 6 Conclusions

In summary, OO use reduces the incidence of T2D, CVD, and pressure ulcer, and CVD mortality and all‐cause mortality (GRADE: moderate). Notably, OO consumption cuts down the level of HbA1c and FBS in T2D population (GRADE: moderate), insulin and HOMA‐IR (GRADE: high). For every 25 and 10 g/day increase in OO consumption, there is a 22% reduction in incidence or mortality of T2D and 0.22 mg/dL increase in HDL‐c. The beneficial intake dosage is 25 g/day or so, but the duration is not determined in our review. The other associations are uncertain because the quality of 77% of the evidence was very low or low. Since the health mechanisms of OO involving the synergistic effects of various components, disentangling the effects of oleic acid from its interaction with polyphenols and other minor components remains a significant challenge. More prospective studies with high quality about dosage, duration and types of OO, lifestyles, and different populations are warranted to validate these findings and provide a basis for precise recommendations.

## Author Contributions

**Xinrui Zou:** data curation (equal), formal analysis (equal), investigation (lead), software (lead), visualization (lead), writing – original draft (lead), writing – review and editing (lead). **Hong Liu:** data curation (equal), project administration (lead), supervision (lead), writing – review and editing (supporting).

## Ethics Statement

The authors have nothing to report.

## Consent

The authors have nothing to report.

## Conflicts of Interest

The authors declare no conflicts of interest.

## Supporting information


**Data S1:** fsn371115‐sup‐0001‐Supinfo1.docx.

**Data S2:** fsn371115‐sup‐0002‐Supinfo2.docx.

**Data S3:** fsn371115‐sup‐0003‐Supinfo3.docx.

## Data Availability

Data sharing is not applicable to this article as no datasets were generated or analyzed during the current study.
