# Supplementary material for: Cardiovascular, Metabolic and Endocrine, Cancer, Mortality, Derma, and Other Outcomes of Olive Oil and Oleic Acid: An Umbrella Review
Source: Food Sci Nutr. 2025 Oct 22;13(10):e71115. doi: 10.1002/fsn3.71115 (PMC12547079; doi:10.1002/fsn3.71115)
Supplement: Supplementary file 2 — Data S2: fsn371115‐sup‐0002‐Supinfo2.docx. [file FSN3-13-e71115-s001.docx]

**Supplemental table 1.** 16 items of AMSTAR 2 on endocrine and metabolic outcomes.

| **Outcomes** | **Study** | **Item1** | **Item2** | **Item3** | **Item4** | **Item5** | **Item6** | **Item7** | **Item8** | **Item9** | **Item10** | **Item11** | **Item12** | **Item13** | **Item14** | **Item15** | **Item16** | **AMSTAR2** |
| --- | --- | --- | --- | --- | --- | --- | --- | --- | --- | --- | --- | --- | --- | --- | --- | --- | --- | --- |
| risk of T2D mellitus | L Schwingshackl et al. 2017 | Yes | Yes | Yes | Partial yes | Yes | Yes | Partial yes | Yes | Yes | NO | Yes | NO | Yes | Yes | NO | Yes | Low |
| HbA1c(%) | L Schwingshackl et al. 2017 | Yes | Yes | Yes | Partial yes | Yes | Yes | Partial yes | Yes | Yes | NO | Yes | NO | Yes | Yes | Yes | Yes | Moderate |
| Fasting glucose | L Schwingshackl et al. 2017 | Yes | Yes | Yes | Partial yes | Yes | Yes | Partial yes | Yes | Yes | NO | Yes | NO | Yes | Yes | Yes | Yes | Moderate |
| fasting blood glucose | Mojgan Morvaridzadeh et al. 2023 | Yes | Yes | Yes | Partial yes | Yes | Yes | No | Yes | Yes | NO | Yes | Yes | Yes | Yes | Yes | Yes | Low |
| insulin | Mojgan Morvaridzadeh et al. 2023 | Yes | Yes | Yes | Partial yes | Yes | Yes | No | Yes | Yes | NO | Yes | Yes | Yes | Yes | Yes | Yes | Low |
| HOMA-IR level | Mojgan Morvaridzadeh et al. 2023 | Yes | Yes | Yes | Partial yes | Yes | Yes | No | Yes | Yes | NO | Yes | Yes | Yes | Yes | Yes | Yes | Low |
| total T2D (incidence and mortality combined) | Manuela Neuenschwander et al. 2023 | Yes | Yes | Yes | Partial yes | Yes | Yes | Partial yes | Yes | Yes | NO | Yes | Yes | Yes | Yes | No | Yes | Low |
| T2D(incidence or mortality) | Miguel A. Martínez-Gonz alez et al. 2022 | Yes | Yes | Yes | Partial yes | Yes | Yes | Yes | Yes | Yes | NO | Yes | Yes | Yes | Yes | NO | Yes | Low |
| 117 factors of MetS | Rosario Pastor et al. 2021 | Yes | Yes | Yes | Partial yes | Yes | Yes | Partial yes | Yes | Yes | NO | Yes | NO | Yes | Yes | NO | Yes | Low |
| glycemic profile | Rosario Pastor et al. 2021 | Yes | Yes | Yes | Partial yes | Yes | Yes | Partial yes | Yes | Yes | NO | Yes | NO | Yes | Yes | NO | Yes | Low |
| 246 factors of MetS | Rosario Pastor et al. 2021 | Yes | Yes | Yes | Partial yes | Yes | Yes | Partial yes | Yes | Yes | NO | Yes | NO | Yes | Yes | NO | Yes | Low |
| glycemic profile | Rosario Pastor et al. 2021 | Yes | Yes | Yes | Partial yes | Yes | Yes | Partial yes | Yes | Yes | NO | Yes | NO | Yes | Yes | NO | Yes | Low |

**Supplemental table 2.** 16 items of AMSTAR 2 on anthropometric indices outcomes.

| **Outcomes** | **Study** | **Item1** | **Item2** | **Item3** | **Item4** | **Item5** | **Item6** | **Item7** | **Item8** | **Item9** | **Item10** | **Item11** | **Item12** | **Item13** | **Item14** | **Item15** | **Item16** | **AMSTAR2** |
| --- | --- | --- | --- | --- | --- | --- | --- | --- | --- | --- | --- | --- | --- | --- | --- | --- | --- | --- |
| BMI(kg/m2) | Ana Clara Neville Armond Santos et al. 2023 | Yes | Yes | Yes | Partial yes | Yes | Yes | Partial yes | Yes | Yes | NO | Yes | Yes | Yes | Yes | Yes | Yes | High |
| waist circumference(cm) |  | Yes | Yes | Yes | Partial yes | Yes | Yes | Partial yes | Yes | Yes | NO | Yes | Yes | Yes | Yes | Yes | Yes | High |
| waist circumference(cm) |  | Yes | Yes | Yes | Partial yes | Yes | Yes | Partial yes | Yes | Yes | NO | Yes | Yes | Yes | Yes | Yes | NO | low |
| waist circumference(cm) |  | Yes | Yes | Yes | Partial yes | Yes | Yes | Partial yes | Yes | Yes | NO | Yes | Yes | Yes | Yes | Yes | NO | low |
| hip circumference(cm) |  | Yes | Yes | Yes | Partial yes | Yes | Yes | Partial yes | Yes | Yes | NO | Yes | Yes | Yes | Yes | Yes | Yes | High |
| waist to hip ratio |  | Yes | Yes | Yes | Partial yes | Yes | Yes | Partial yes | Yes | Yes | NO | Yes | Yes | Yes | Yes | Yes | Yes | High |
| mean total body fat(kg) |  | Yes | Yes | Yes | Partial yes | Yes | Yes | Partial yes | Yes | Yes | NO | Yes | Yes | Yes | Yes | Yes | Yes | High |
| mean total body fat(kg) |  | Yes | Yes | Yes | Partial yes | Yes | Yes | Partial yes | Yes | Yes | NO | Yes | Yes | Yes | Yes | Yes | No | low |
| mean total body fat(kg) |  | Yes | Yes | Yes | Partial yes | Yes | Yes | Partial yes | Yes | Yes | NO | Yes | Yes | Yes | Yes | Yes | No | low |
| mean total body fat/adipose mass(%) |  | Yes | Yes | Yes | Partial yes | Yes | Yes | Partial yes | Yes | Yes | NO | Yes | Yes | Yes | Yes | Yes | Yes | High |
| mean mussle mass/lean mass(kg) |  | Yes | Yes | Yes | Partial yes | Yes | Yes | Partial yes | Yes | Yes | NO | Yes | Yes | Yes | Yes | Yes | Yes | High |
| mean mussle mass/lean mass(kg) |  | Yes | Yes | Yes | Partial yes | Yes | Yes | Partial yes | Yes | Yes | NO | Yes | Yes | Yes | Yes | Yes | NO | Low |
| mean mussle mass/lean mass(kg) |  | Yes | Yes | Yes | Partial yes | Yes | Yes | Partial yes | Yes | Yes | NO | Yes | Yes | Yes | Yes | Yes | NO | low |
| weight | Mojgan Morvaridzadeh et al. 2023 | Yes | Yes | Yes | Partial yes | Yes | Yes | Partial yes | Yes | Yes | NO | Yes | Yes | Yes | Yes | Yes | Yes | High |
| BMI(kg/m2) | Mojgan Morvaridzadeh et al. 2023 | Yes | Yes | Yes | Partial yes | Yes | Yes | Partial yes | Yes | Yes | NO | Yes | Yes | Yes | Yes | Yes | Yes | High |
| WC | Mojgan Morvaridzadeh et al. 2023 | Yes | Yes | Yes | Partial yes | Yes | Yes | Partial yes | Yes | Yes | NO | Yes | Yes | Yes | Yes | Yes | Yes | High |
| WHR | Mojgan Morvaridzadeh et al. 2023 | Yes | Yes | Yes | Partial yes | Yes | Yes | Partial yes | Yes | Yes | NO | Yes | Yes | Yes | Yes | No | Yes | Low |
| body composition | Rosario Pastor et al. 2021 | Yes | Yes | Yes | Partial yes | Yes | Yes | Partial yes | Yes | Yes | NO | Yes | NO | Yes | Yes | NO | Yes | low |

**Supplemental table 3.** 16 items of AMSTAR 2 on inflammation markers.

| **Outcomes** | **Study** | **Item1** | **Item2** | **Item3** | **Item4** | **Item5** | **Item6** | **Item7** | **Item8** | **Item9** | **Item10** | **Item11** | **Item12** | **Item13** | **Item14** | **Item15** | **Item16** | **AMSTAR2** |
| --- | --- | --- | --- | --- | --- | --- | --- | --- | --- | --- | --- | --- | --- | --- | --- | --- | --- | --- |
| lipoprotein a | Mojgan Morvaridzadeh et al. 2023 | Yes | Yes | Yes | Partial yes | Yes | Yes | Partial yes | Yes | Yes | NO | Yes | Yes | Yes | Yes | No | Yes | Low |
| CRP | Mojgan Morvaridzadeh et al. 2023 | Yes | Yes | Yes | Partial yes | Yes | Yes | Partial yes | Yes | Yes | NO | Yes | Yes | Yes | Yes | Yes | Yes | High |
| IL-6 | Mojgan Morvaridzadeh et al. 2023 | Yes | Yes | Yes | Partial yes | Yes | Yes | Partial yes | Yes | Yes | NO | Yes | Yes | Yes | Yes | No | Yes | Low |
| IL-10 | Mojgan Morvaridzadeh et al. 2023 | Yes | Yes | Yes | Partial yes | Yes | Yes | Partial yes | Yes | Yes | NO | Yes | Yes | Yes | Yes | No | Yes | Low |
| TNF-α | Mojgan Morvaridzadeh et al. 2023 | Yes | Yes | Yes | Partial yes | Yes | Yes | Partial yes | Yes | Yes | NO | Yes | Yes | Yes | Yes | No | Yes | Low |
| CRP | Lukas Schwingshackl et al.2015 | Yes | Yes | Yes | Partial yes | Yes | Yes | NO | Yes | Yes | NO | Yes | NO | Yes | Yes | Yes | Yes | low |
| IL-6 | Lukas Schwingshackl et al.2015 | Yes | Yes | Yes | Partial yes | Yes | Yes | NO | Yes | Yes | NO | Yes | NO | Yes | Yes | NO | Yes | Critically low |
| FMD% | Lukas Schwingshackl et al.2015 | Yes | Yes | Yes | Partial yes | Yes | Yes | NO | Yes | Yes | NO | Yes | NO | Yes | Yes | NO | Yes | Critically low |
| Adiponectin | Lukas Schwingshackl et al.2015 | Yes | Yes | Yes | Partial yes | Yes | Yes | NO | Yes | Yes | NO | Yes | NO | Yes | Yes | NO | Yes | Critically low |
| TNF-α | Lukas Schwingshackl et al.2015 | Yes | Yes | Yes | Partial yes | Yes | Yes | NO | Yes | Yes | NO | Yes | NO | Yes | Yes | NO | Yes | Critically low |
| sE-Selectin | Lukas Schwingshackl et al.2015 | Yes | Yes | Yes | Partial yes | Yes | Yes | NO | Yes | Yes | NO | Yes | NO | Yes | Yes | NO | Yes | Critically low |
| sP-Selectin | Lukas Schwingshackl et al.2015 | Yes | Yes | Yes | Partial yes | Yes | Yes | NO | Yes | Yes | NO | Yes | NO | Yes | Yes | NO | Yes | Critically low |
| sICAM-1 | Lukas Schwingshackl et al.2015 | Yes | Yes | Yes | Partial yes | Yes | Yes | NO | Yes | Yes | NO | Yes | NO | Yes | Yes | NO | Yes | Critically low |
| sVCAM-1 | Lukas Schwingshackl et al.2015 | Yes | Yes | Yes | Partial yes | Yes | Yes | NO | Yes | Yes | NO | Yes | NO | Yes | Yes | NO | Yes | Critically low |

**Supplemental table 4.** 16 items of AMSTAR 2 on cardiovascular diseases.

| **Outcomes** | **Study** | **Item1** | **Item2** | **Item3** | **Item4** | **Item5** | **Item6** | **Item7** | **Item8** | **Item9** | **Item10** | **Item11** | **Item12** | **Item13** | **Item14** | **Item15** | **Item16** | **AMSTAR2** |
| --- | --- | --- | --- | --- | --- | --- | --- | --- | --- | --- | --- | --- | --- | --- | --- | --- | --- | --- |
| SBP | Zamora-Zamora, F. et al. 2018 | Yes | Partial yes | Yes | Partial yes | Yes | Yes | Partial yes | Yes | Yes | NO | NO | Yes | Yes | Yes | Yes | NO | Low |
| DBP |  | Yes | Partial yes | Yes | Partial yes | Yes | Yes | Partial yes | Yes | Yes | NO | NO | Yes | Yes | Yes | Yes | NO | Low |
| SBP | Mojgan Morvaridzadeh et al. 2023 | Yes | Yes | Yes | Partial yes | Yes | Yes | Partial yes | Yes | Yes | NO | Yes | Yes | Yes | Yes | No | Yes | Low |
| DBP |  | Yes | Yes | Yes | Partial yes | Yes | Yes | Partial yes | Yes | Yes | NO | Yes | Yes | Yes | Yes | Yes | Yes | High |
| total CVD(incidence of CVD, CHD, MI, and CVD mortality) | Manuela Neuenschwander et al. 2023 | Yes | Yes | Yes | Partial yes | Yes | Yes | Partial yes | Yes | Yes | NO | Yes | Yes | Yes | Yes | No | Yes | Low |
| CVD mortality |  | Yes | Yes | Yes | Partial yes | Yes | Yes | Partial yes | Yes | Yes | NO | Yes | Yes | Yes | Yes | No | Yes | Low |
| CVD | Miguel A. Martínez-Gonzalez et al. 2022 | Yes | Yes | Yes | Partial yes | Yes | Yes | Yes | Yes | Yes | NO | Yes | Yes | Yes | Yes | Yes | Yes | High |
| CVD(CHD and stroke) | Martinez-Gonzalez, M. A. et al.2014 | Yes | Partial yes | Yes | Partial yes | Yes | Yes | Partial yes | Yes | Yes | NO | Yes | Yes | Yes | Yes | Yes | Yes | High |
| CHD |  | Yes | Partial yes | Yes | Partial yes | Yes | Yes | Partial yes | Yes | Yes | NO | Yes | Yes | Yes | Yes | Yes | Yes | High |
| Stroke |  | Yes | Partial yes | Yes | Partial yes | Yes | Yes | Partial yes | Yes | Yes | NO | Yes | Yes | Yes | Yes | Yes | Yes | High |
| Cardiovascular mortality | Lukas Schwingshackl et al. 2014 | Yes | Partial yes | Yes | Partial yes | Yes | NO | NO | Yes | Yes | NO | Yes | Yes | Yes | Yes | Yes | NO | low |
| Combined cardiovascular events |  | Yes | Partial yes | Yes | Partial yes | Yes | NO | NO | Yes | Yes | NO | Yes | Yes | Yes | Yes | Yes | NO | low |
| Coronary heart disease |  | Yes | Partial yes | Yes | Partial yes | Yes | NO | NO | Yes | Yes | NO | Yes | Yes | Yes | Yes | Yes | NO | low |
| Stroke |  | Yes | Partial yes | Yes | Partial yes | Yes | NO | NO | Yes | Yes | NO | Yes | Yes | Yes | Yes | Yes | NO | low |

**Supplemental table 5.** 16 items of AMSTAR 2 on blood lipids.

| **Outcomes** | **Study** | **Item1** | **Item2** | **Item3** | **Item4** | **Item5** | **Item6** | **Item7** | **Item8** | **Item9** | **Item10** | **Item11** | **Item12** | **Item13** | **Item14** | **Item15** | **Item16** | **AMSTAR2** |
| --- | --- | --- | --- | --- | --- | --- | --- | --- | --- | --- | --- | --- | --- | --- | --- | --- | --- | --- |
| TG | Mojgan Morvaridzadeh et al. 2023 | Yes | Yes | Yes | Partial yes | Yes | Yes | Partial yes | Yes | Yes | NO | Yes | Yes | Yes | Yes | Yes | Yes | High |
| TC | Mojgan Morvaridzadeh et al. 2023 | Yes | Yes | Yes | Partial yes | Yes | Yes | Partial yes | Yes | Yes | NO | Yes | Yes | Yes | Yes | Yes | Yes | High |
| LDL | Mojgan Morvaridzadeh et al. 2023 | Yes | Yes | Yes | Partial yes | Yes | Yes | Partial yes | Yes | Yes | NO | Yes | Yes | Yes | Yes | Yes | Yes | High |
| HDL | Mojgan Morvaridzadeh et al. 2023 | Yes | Yes | Yes | Partial yes | Yes | Yes | Partial yes | Yes | Yes | NO | Yes | Yes | Yes | Yes | Yes | Yes | High |
| VLDL | Mojgan Morvaridzadeh et al. 2023 | Yes | Yes | Yes | Partial yes | Yes | Yes | Partial yes | Yes | Yes | NO | Yes | Yes | Yes | Yes | No | Yes | Low |
| ApoA-I | Mojgan Morvaridzadeh et al. 2023 | Yes | Yes | Yes | Partial yes | Yes | Yes | Partial yes | Yes | Yes | NO | Yes | Yes | Yes | Yes | Yes | Yes | High |
| ApoB | Mojgan Morvaridzadeh et al. 2023 | Yes | Yes | Yes | Partial yes | Yes | Yes | Partial yes | Yes | Yes | NO | Yes | Yes | Yes | Yes | No | Yes | Low |
| lipid profile | Rosario Pastor et al. 2021 | Yes | Yes | Yes | Partial yes | Yes | Yes | Partial yes | Yes | Yes | NO | Yes | NO | Yes | Yes | NO | Yes | Low |
| lipid profile | Rosario Pastor et al. 2021 | Yes | Yes | Yes | Partial yes | Yes | Yes | Partial yes | Yes | Yes | NO | Yes | NO | Yes | Yes | NO | Yes | Low |
| postprandial triglycerides over 8h | Milena Monfort-Pires et al.2016 | Yes | Partial yes | Yes | No | Yes | Yes | Yes | Yes | Yes | No | Yes | Yes | Yes | Yes | Yes | Yes | low |
| postprandial triglycerides over 4h | Milena Monfort-Pires et al.2016 | Yes | Partial yes | Yes | No | Yes | Yes | Yes | Yes | Yes | No | Yes | Yes | Yes | Yes | Yes | Yes | Low |
| postprandial triglycerides over 6h | Milena Monfort-Pires et al.2016 | Yes | Partial yes | Yes | No | Yes | Yes | Yes | Yes | Yes | No | Yes | Yes | Yes | Yes | Yes | Yes | low |
| TC | Jabbarzadeh-Ganjeh, Bahareh et al.2023 | Yes | Yes | Yes | Partial yes | Yes | Yes | Yes | Yes | Yes | No | Yes | Yes | Yes | Yes | Yes | Yes | High |
| LDL | Jabbarzadeh-Ganjeh, Bahareh et al.2023 | Yes | Yes | Yes | Partial yes | Yes | Yes | Yes | Yes | Yes | No | Yes | Yes | Yes | Yes | Yes | Yes | High |
| HDL | Jabbarzadeh-Ganjeh, Bahareh et al.2023 | Yes | Yes | Yes | Partial yes | Yes | Yes | Yes | Yes | Yes | No | Yes | Yes | Yes | Yes | Yes | Yes | High |
| TAG | Jabbarzadeh-Ganjeh, Bahareh et al.2023 | Yes | Yes | Yes | Partial yes | Yes | Yes | Yes | Yes | Yes | No | Yes | Yes | Yes | Yes | Yes | Yes | High |

**Supplemental table 6.** 16 items of AMSTAR 2 on all-cause mortality.

| **Outcomes** | **Study** | **Item1** | **Item2** | **Item3** | **Item4** | **Item5** | **Item6** | **Item7** | **Item8** | **Item9** | **Item10** | **Item11** | **Item12** | **Item13** | **Item14** | **Item15** | **Item16** | **AMSTAR2** |
| --- | --- | --- | --- | --- | --- | --- | --- | --- | --- | --- | --- | --- | --- | --- | --- | --- | --- | --- |
| all-cause mortality | Miguel A. Martínez-Gonzalez et al. 2022 | Yes | Yes | Yes | Partial yes | Yes | Yes | Yes | Yes | Yes | NO | Yes | Yes | Yes | Yes | Yes | Yes | High |
| All-cause mortality | Lukas Schwingshackl et al. 2014 | Yes | Partial yes | Yes | Partial yes | Yes | NO | NO | Yes | Yes | NO | Yes | Yes | Yes | Yes | Yes | NO | low |

.

**Supplemental table 6.** 16 items of AMSTAR 2 on cancer outcomes

| **Outcomes** | **Study** | **Item1** | **Item2** | **Item3** | **Item4** | **Item5** | **Item6** | **Item7** | **Item8** | **Item9** | **Item10** | **Item11** | **Item12** | **Item13** | **Item14** | **Item15** | **Item16** | **AMSTAR2** |
| --- | --- | --- | --- | --- | --- | --- | --- | --- | --- | --- | --- | --- | --- | --- | --- | --- | --- | --- |
| Cancer (incidence or mortality) | Miguel A. Martínez-Gonzalez et al. 2022 | Yes | Yes | Yes | Partial yes | Yes | Yes | Yes | Yes | Yes | NO | Yes | Yes | Yes | Yes | Yes | Yes | High |
| Risk for  Overall cancer | Christos Markellos et al.2022 | Yes | Partial yes | No | Partial yes | Yes | Yes | Yes | Yes | Yes | NO | Yes | NO | NO | Yes | Yes | Yes | Low |
| Breast cancer  risk | Naria Sealy et al.2020 | Yes | Partial yes | Yes | Partial yes | Yes | No | Partial Yes | Yes | Yes | NO | Yes | NO | Yes | Yes | Yes | Yes | Moderate |
| Gastrointestina  l cancer | Christos Markellos et al.2022 | Yes | Partial yes | No | Partial yes | Yes | Yes | Yes | Yes | Yes | NO | Yes | NO | NO | Yes | Yes | Yes | Low |
| Upper  aerodigestive  cancers | Christos Markellos et al.2022 | Yes | Partial yes | No | Partial yes | Yes | Yes | Yes | Yes | Yes | NO | Yes | NO | NO | Yes | Yes | Yes | Low |
| Urinary tract cancers | Christos Markellos et al.2022 | Yes | Partial yes | No | Partial yes | Yes | Yes | Yes | Yes | Yes | NO | Yes | NO | NO | Yes | Yes | Yes | Low |
| Colorectal  cancer | Christos Markellos et al.2022 | Yes | Partial yes | No | Partial yes | Yes | Yes | Yes | Yes | Yes | NO | Yes | NO | NO | Yes | Yes | Yes | Low |
| Esophageal  cancer | Christos Markellos et al.2022 | Yes | Partial yes | No | Partial yes | Yes | Yes | Yes | Yes | Yes | NO | Yes | NO | NO | Yes | Yes | Yes | Low |
| Gastric  cancer | Christos Markellos et al.2022 | Yes | Partial yes | No | Partial yes | Yes | Yes | Yes | Yes | Yes | NO | Yes | NO | NO | Yes | Yes | Yes | Low |
| Prostate  cancer | Christos Markellos et al.2022 | Yes | Partial yes | No | Partial yes | Yes | Yes | Yes | Yes | Yes | NO | Yes | NO | NO | Yes | Yes | Yes | Low |

**Supplemental table 7.** 16 items of AMSTAR 2 on derma application outcomes

| **Outcomes** | **Study** | **Item1** | **Item2** | **Item3** | **Item4** | **Item5** | **Item6** | **Item7** | **Item8** | **Item9** | **Item10** | **Item11** | **Item12** | **Item13** | **Item14** | **Item15** | **Item16** | **AMSTAR2** |
| --- | --- | --- | --- | --- | --- | --- | --- | --- | --- | --- | --- | --- | --- | --- | --- | --- | --- | --- |
| Incidence of RTOG grade 1–2 | Jolien Robijns et al.2023 | Yes | Partial yes | Yes | Partial yes | Yes | Yes | Partial yes | Yes | Yes | Yes | Yes | NO | Yes | Yes | NO | Yes | Low |
| RTOG grade 2+ | Jolien Robijns et al.2023 | Yes | Partial yes | Yes | Partial yes | Yes | No | Partial yes | Yes | Yes | Yes | Yes | NO | Yes | Yes | NO | Yes | Low |
| RTOG grade 3+ | Jolien Robijns et al.2023 | Yes | Partial yes | Yes | Partial yes | Yes | Yes | Partial yes | Yes | Yes | Yes | Yes | NO | Yes | Yes | NO | Yes | Low |
| Incidence of Pressure Ulcers | Akram Hernández-Vásquez et al.2022 | Yes | Yes | Yes | Yes | Yes | Yes | Yes | Yes | Yes | Yes | Yes | Yes | Yes | Yes | No | Yes | Low |
| Adverse Events | Akram Hernández-Vásquez et al.2022 | Yes | Yes | Yes | Yes | Yes | Yes | Yes | Yes | Yes | Yes | Yes | Yes | Yes | Yes | No | Yes | Low |

**Supplemental table 8.** 16 items of AMSTAR 2 on other outcomes

| **Outcomes** | **Study** | **Item1** | **Item2** | **Item3** | **Item4** | **Item5** | **Item6** | **Item7** | **Item8** | **Item9** | **Item10** | **Item11** | **Item12** | **Item13** | **Item14** | **Item15** | **Item16** | **AMSTAR2** |
| --- | --- | --- | --- | --- | --- | --- | --- | --- | --- | --- | --- | --- | --- | --- | --- | --- | --- | --- |
| maternal-fetal outcomes (SGA and LGA newborns, GDM, preeclampsia, and cardiovascular risk) | Anna Carolina Cortez-Ribeiro et al. 2023 | Yes | Partial yes | Yes | Partial yes | Yes | Yes | Yes | Yes | Yes | NO | NO | NO | Yes | Yes | NO | Yes | Critically low |
| cognitive fuction in the elder adults | Asra Fazlollahi et al. 2023 | Yes | Partial yes | Yes | Partial yes | Yes | Yes | Partial yes | Yes | Yes | NO | NO | NO | Yes | Yes | NO | Yes | Critically low |
| hepatic steatosis | Yiwei Ma et al. 2023 | Yes | Yes | Yes | Yes | Yes | Yes | Yes | Yes | Yes | NO | NO | NO | Partial yes | Yes | NO | Yes | Critically low |
| liver enzymes | Yiwei Ma et al. 2023 | Yes | Yes | Yes | Yes | Yes | Yes | Yes | Yes | Yes | NO | NO | NO | Partial yes | Yes | NO | Yes | Critically low |
