# Supplementary material for: Cardiovascular, Metabolic and Endocrine, Cancer, Mortality, Derma, and Other Outcomes of Olive Oil and Oleic Acid: An Umbrella Review
Source: Food Sci Nutr. 2025 Oct 22;13(10):e71115. doi: 10.1002/fsn3.71115 (PMC12547079; doi:10.1002/fsn3.71115)
Supplement: Supplementary file 3 — Data S3: fsn371115‐sup‐0003‐Supinfo3.docx. [file FSN3-13-e71115-s002.docx]

**Supplemental table1.** Characteristics of studies included in the endocrine and metabolic outcomes

| **Outcomes** | **Studies** | **Population** | **Intervention** | **Comparison** |
| --- | --- | --- | --- | --- |
| risk of T2D mellitus | L Schwingshackl et al. 2017 | NHS, NHS II, EPIC-InterAct, SUN, male and female,18-80 years | **FFQ** for olive oil intake;5.7-22 years follow up | High vs low olive oil |
| HbA1c(%) | L Schwingshackl et al. 2017 | T2D patients (51-67 years); Mean baseline HbA1c (5.2-11.3%);Mean baseline BMI（25-31 kg/m²） | Dietary olive oil: 10-118 g/day (mostly 15-50g daily); capsule(2-4g/d); olive oil as high MUFA(30-75g/d); 2w-48m(mostly 3.5w-6m) | Olive oil vs low-fat diet; Olive oil vs polyunsaturated fatty acids (PUFA)-rich oils; Olive oil vs fish oil |
| Fasting glucose | L Schwingshackl et al. 2017 | T2D patients (51-67 years); Mean baseline FBG(6.6-11.2  mmol/L） | as above | Olive oil vs low-fat diet; Olive oil vs polyunsaturated fatty acids (PUFA)-rich oils; Olive oil vs fish oil |
| fasting blood glucose | Mojgan Morvaridzadeh et al. 2023 | Healthy population,T2D,CHD, Non-alcoholic fatty liver disease， Metabolic syndrome， obese population | EVOO,10-60ml/d, 16-20%TE | Sunflower oil, Rice bran oil, Palm oil, Refined olive oil, Regular diet |
| insulin | Mojgan Morvaridzadeh et al. 2023 | Healthy population,T2D, Metabolic syndrome， obese population | EVOO,20-54g/d, 16-30%TE | Corn oil,Palm oil, Refined olive oil, Regular diet |
| HOMA-IR level | Mojgan Morvaridzadeh et al. 2023 | Healthy population,T2D, Non-alcoholic fatty liver disease， Metabolic syndrome | EVOO,20-30g/d, 20%TE | Sunflower oil, Rice bran oil, Palm oil, Regular diet |
| total T2D (incidence and mortality combined) | Manuela Neuenschwander et al. 2023 | NIH-AARP and Healthy study, NHS, HPFS | 5g olive oil | 5g butter |
| T2D(incidence or mortality) | Miguel A. Martínez-Gonz alez et al. 2022 | SUN, NHS, HPFS, NIH-AARP cohort; PREDIMED | Unspecific Olive oil consumption; Mediterranean Diet+EVOO; FFQ | Lowest olive oil intake; low fat diet |
| 117 factors of MetS | Rosario Pastor et al. 2021 | Healthy population, overweight, metabolic syndrome, hypertension, hypercholesterolemia;18-over 70 years old, male and female  - | 4.5g OA; High-oleic sunflower oil(or capsule), High-oleic canola oil, 56g High-oleic peanuts, Olive oil, 200ml milk (3g OA), ground beef patties; 15-25% TE; 3-24w(mostly 4-12w)  - | PUFA; normal peanuts; SFA; Habitual Diet; Placebo  - |
| glycemic profile | Rosario Pastor et al. 2021 |  |  |  |
| 246 factors of MetS | Rosario Pastor et al. 2021 | Non-alcoholic fatty liver disease (NAFLD),Dyslipidemia, Metabolic syndrome, Type 2 diabetes, Hypertension, Hypercholesterolemia patients  - | Olive oil liquid form(10-54g/d), capsule(2-4g/d); Mediterranean Diet with EVOO; olive oil for cooking; low fat diet with olive oil  - | PUFA, SFA, MUFA; supplement with Plant Sterols or Phospholipids or MCT Oil or CLA; low fat diet with high carbohydrate,regular diet  - |
| glycemic profile | Rosario Pastor et al. 2021 |  |  |  |

**Supplemental table2.** Characteristics of studies included in the anthropometric indices outcomes.

| **Outcomes** | **Studies** | **Population** | **Intervention** | **Comparison** |
| --- | --- | --- | --- | --- |
| BMI(kg/m2) | Ana Clara Neville Armond Santos et al. 2023 | Overweight or obesity individuals；high risk of cardiovascular disease；healthy adults；elderly | Culinary olive oil (18-128.5 g/d); Capsule (mostly 0.43-5g/d or 9g/d); 4-240weeks | Soybean oil, sunflower seed oil, canola oil, palm oil, coconut oil, cottonseed oil, flaxseed oil, Low-fat diet, nut supplementation (as part of the MedDiet), traditional Brazilian diet, CLA, fish oil, placebo capsules |
| waist circumference(cm) | Ana Clara Neville Armond Santos et al. 2023 | Overweight or obesity individuals；high risk of cardiovascular disease (Metabolic syndrome, diabetes, hypercholesterolemia)  Overweight or obesity individuals (adults or elderly); healthy adults | Olive oil and EVOO  Culinary olive oil (20-50 g/d); mostly 8-24w or >1 year  Capsule (mostly 0.43-5g/d or 9g/d) | CLA (conjugated linoleic acid), fish oil, sunflower seed oil, canola oil |
| waist circumference(cm) |  |  |  |  |
| waist circumference(cm) | Ana Clara Neville Armond Santos et al. 2023 |  |  |  |
| hip circumference(cm) | Ana Clara Neville Armond Santos et al. 2023 |  |  |  |
| waist to hip ratio | Ana Clara Neville Armond Santos et al. 2023 |  |  |  |
| mean total body fat(kg) | Ana Clara Neville Armond Santos et al. 2023 |  |  |  |
| mean total body fat(kg) | Ana Clara Neville Armond Santos et al. 2023 |  |  |  |
| mean total body fat(kg) | Ana Clara Neville Armond Santos et al. 2023 |  |  |  |
| mean total body fat/adipose mass(%) | Ana Clara Neville Armond Santos et al. 2023 |  |  |  |
| mean mussle mass/lean mass(kg) | Ana Clara Neville Armond Santos et al. 2023 |  |  |  |
| mean mussle mass/lean mass(kg) | Ana Clara Neville Armond Santos et al. 2023 |  |  |  |
| mean mussle mass/lean mass(kg) | Ana Clara Neville Armond Santos et al. 2023 |  |  |  |
| weight | Mojgan Morvaridzadeh et al. 2023 | Healthy population,T2D, Non-alcoholic fatty liver disease， obese population | EVOO,20-50ml/d | Cooking oil, Regular diet |
| BMI(kg/m2) | Mojgan Morvaridzadeh et al. 2023 |  |  |  |
| WC | Mojgan Morvaridzadeh et al. 2023 | Healthy population,T2D, Metabolic syndrome， obese population,depression, | EVOO,20-30g/d | Sunflower oil, Regular diet |
| WHR | Mojgan Morvaridzadeh et al. 2023 |  |  |  |
| body composition | Rosario Pastor et al. 2021 | Healthy population, overweight, metabolic syndrome, hypertension, hypercholesterolemia;18-over 70 years old, male and female | 4.5g OA; High-oleic sunflower oil(or capsule), High-oleic canola oil, 56g High-oleic peanuts, Olive oil, 200ml milk (3g OA), ground beef patties; 15-25% TE; 3-24w(mostly 4-12w) | PUFA; normal peanuts; SFA; Habitual Diet; Placebo |

**Supplemental table3.** Characteristics of studies included in the inflammation marker outcomes.

| **Outcomes** | **Study** | **Population** | **Intervention** | **Comparison** |
| --- | --- | --- | --- | --- |
| lipoprotein a | Mojgan Morvaridzadeh et al. 2023 | Healthy population,T2D, Metabolic syndrome，depression, Fibromyalgia patient | EVOO,25-50ml/d;  2-12weeks, mostly 6-12weeeks | Sunflpwer oil, refined olive oil, regular diet |
| CRP | Mojgan Morvaridzadeh et al. 2023 |  |  |  |
| IL-6 | Mojgan Morvaridzadeh et al. 2023 | Healthy population, obese population, CHD | EVOO,25-54g/d;  2-12weeks, mostly 6-12weeeks | Palm oil, soybean oil, Refined olive oil, regular diet |
| IL-10 | Mojgan Morvaridzadeh et al. 2023 |  |  |  |
| TNF-α | Mojgan Morvaridzadeh et al. 2023 |  |  |  |
| CRP | Lukas Schwingshackl et al.2015 | Healthy individuals, overweight/obese, people with type 2 diabetes, individuals at high cardiovascular risk, chronic heart failure, NAFLD, breast cancer survivors. Age: 23-68 years. BMI: 21.9-32.6 kg/m². | Forms: Capsules (1-6 g/day), liquid oil (as part of MedD: 30-50 mL/day; or separate supplementation: 5-50 mL/day). Types: Extra virgin, virgin, and refined olive oils are all available.4w-4y, mostly 6-12w | • Other oil capsules: Fish oil/DHA/EPA, CLA, krill oil, safflower oil• Different dietary patterns: Low-fat diet, MedD nuts, Western diet, NCI diet, high SFA diet• Other liquid oils: Flaxseed oil, coconut oil, palm oil |
| IL-6 | Lukas Schwingshackl et al.2015 | Diverse: Healthy, Abdominally Obese (postmenopausal women), Type 2 Diabetes, Hypertension, Chronic Heart Failure. Age: 39-66 yrs BMI: 23.1-32.6 kg/m² | Form: Primarily capsules (2-5 mL/day or 2-4 g/day), Liquid oil (as part of MedD) Type: Extra Virgin, Refined olive oil, 6 - 24 weeks Common: 6-12 weeks | Primarily other oil capsules: • Fish oil/EPA/DHA capsules (most common) • Flaxseed oil (liquid) • CLA capsules |
| FMD% | Lukas Schwingshackl et al.2015 | Primarily patients: Type 2 Diabetes, History of CHD, NAFLD, Overweight/Healthy Age: 23-61 yrs BMI: 24.65-31.3 kg/m² | Form: Capsules (2-4 g/day) and Liquid oil (as part of MedD: 30-50 mL/day) Type: Extra Virgin, Virgin, Refined olive oil. 4 weeks - 1 year (52 wks) Common: 6-12 weeks | • n-3 fatty acid capsules (Fish oil/DHA) • Low-fat diet • CLA capsules • n-3 enriched olive oil |
| Adiponectin | Lukas Schwingshackl et al.2015 | Healthy, Abdominally Obese (postmenopausal women), Type 2 Diabetes, NAFLD. Age: 24.9-66 yrs BMI: 22.9-32.6 kg/m² | Form: Primarily capsules (2-6.5 g/day or 5 mL/day), Liquid oil (as part of MedD) Type: Extra Virgin, Refined olive oil. 6 weeks - 4 years (208 wks)  Common: 6-12 weeks | Primarily other oil capsules:  • Fish oil capsules  • CLA capsules  • n-3 enriched olive oil  • Low-fat diet |
| TNF-α | Lukas Schwingshackl et al.2015 | Healthy or Patients: Healthy, Type 2 Diabetes with Hypertension, Overweight/Healthy Age: 25.6-61.5 yrs BMI: 21.9-29.9 kg/m² | Form: Capsules (2-4 g/day) and Liquid oil (15 mL/day) Type: Extra Virgin, Refined olive oil. 6 weeks | Primarily other oil capsules: • Fish oil/EPA/DHA capsules • Flaxseed oil (liquid) • CLA capsules |
| sE-Selectin | Lukas Schwingshackl et al.2015 | High-risk or Patients: High CVD risk, Type 2 Diabetes, Metabolic Syndrome/CHD, Healthy. Age: 23.3-68.1 yrs BMI: 24.65-28.1 kg/m² | Form: Liquid oil (as part of MedD: 50 mL/day) and Capsules (1-4.5 g/day) Type: Extra Virgin, Refined olive oil. 4 weeks - 52 weeks | • Low-fat diet • CLA capsules • Safflower oil capsules • DHA capsules • High SFA diet |
| sP-Selectin | Lukas Schwingshackl et al.2015 | Patients: High CVD risk, Chronic Heart Failure, Type 2 Diabetes with Hypertension. Age: 56-68.1 yrs BMI: 25.7-31.3 kg/m² | Form: Liquid oil (as part of MedD: 50 mL/day or 1L/week) and Capsules (1 g/day) Type: Extra Virgin, Refined olive oil. 4 weeks - 52 weeks | • Low-fat diet • n-3 PUFA capsules • MedD+nuts • DHA capsules |
| sICAM-1 | Lukas Schwingshackl et al.2015 | Healthy, Chronic Heart Failure, Type 2 Diabetes/High risk, History of CHD, Metabolic Syndrome/CHD. Age: 23.3-66 yrs BMI: 24.65-28.1 kg/m² | Form: Capsules (1-4.5 g/day) and Liquid oil (as part of MedD: 30-50 mL/day) Type: Extra Virgin, Virgin, Refined olive oil. 4 weeks - 52 weeks | • n-3 PUFA capsules • MedD+nuts/almonds • Low-fat diet • CLA/Safflower oil capsules • High SFA diet |
| sVCAM-1 | Lukas Schwingshackl et al.2015 | Healthy, Chronic Heart Failure, Type 2 Diabetes/High risk, History of CHD, Metabolic Syndrome/CHD. Age: 23.3-66 yrs BMI: 24.65-28.1 kg/m² | Form: Capsules (1-4.5 g/day) and Liquid oil (as part of MedD: 30-50 mL/day) Type: Extra Virgin, Virgin, Refined olive oil. 4 weeks - 52 weeks | • n-3 PUFA capsules • MedD+nuts/almonds • Low-fat diet • CLA/Safflower oil capsules • High SFA diet |

**Supplemental table4.** Characteristics of studies included in the cardiovascular diseases outcomes.

| **Outcomes** | **Studies** | **Population** | **Intervention** | **Comparison** |
| --- | --- | --- | --- | --- |
| SBP | Zamora-Zamora, F. et al. 2018 | Healthy adults, overweight or obese individuals, hypertension, type 2 diabetes, diabetic nephropathy or early kidney disease, metabolic syndrome, psoriatic arthritis, pregnant women (twin pregnancy or pregnancy-induced hypertension) | Mostly 3-6 month, 1 or 4 years;EVOO or olive oil, capsule (1-6g/d, liquid 10-50ml/d, or olive oil as a specific dietary pattern (such as the Mediterranean diet, American diet, low-calorie diet) | Sunflower seed oil, corn oil, soybean oil, butter, omega-3 fatty acids (fish oil, DHA/EPA), conjugated linoleic acid (CLA), low-fat diet, regular/habitual diet, capsules (such as microalgae, palmitic acid) |
| DBP | Zamora-Zamora, F. et al. 2018 |  |  |  |
| SBP | Mojgan Morvaridzadeh et al. 2023 | Healthy population,T2D, Metabolic syndrome， obese population | EVOO,10-39g/d | Sunflower oil, corn and soybean oil, Refined olive oil, Butter |
| DBP | Mojgan Morvaridzadeh et al. 2023 |  |  |  |
| total CVD(incidence of CVD, CHD, MI, and CVD mortality) | Manuela Neuenschwander et al. 2023 | NIH-AARP and Healthy study, NHS, HPFS | 5g olive oil | 5g butter |
| CVD mortality | Manuela Neuenschwander et al. 2023 | NIH-AARP and Healthy study, NHS, HPFS | 5g olive oil | 5g butter |
| CVD | Miguel A. Martínez-Gonzalez et al. 2022 | EPIC,SUN, ATTICA ,Three-City, NHS,HPFS, NIH-AARP, ICS, PREDIMED | Unspecific Olive oil consumption; Mediterranean Diet+EVOO; FFQ | Lowest olive oil intake; low fat diet |
| CVD(CHD and stroke) | Martinez-Gonzalez, M. A. et al.2014 | 37%-80% males and females; Mediterranean countries, France, EPIC; 4.8-10.6 years follow up or 4.8-10.4 years follow up | 10-53g/d olive oil; FFQ | 2.6g/d |
| CHD | Martinez-Gonzalez, M. A. et al.2014 |  |  |  |
| Stroke | Martinez-Gonzalez, M. A. et al.2014 |  |  |  |
| Cardiovascular mortality | Lukas Schwingshackl et al. 2014 | USA, Europe, Israel; male and female; adults and elderly | MUFA, MUFA:SFA, olive oil, 4.8-23 years follow up | Lowest intake quantile group |
| Combined cardiovascular events | Lukas Schwingshackl et al. 2014 | USA, Europe, Japan; male and female; youth age-elderly | MUFA, MUFA:SFA, olive oil, oleic acid, 3.7-30 years follow up |  |
| Coronary heart disease | Lukas Schwingshackl et al. 2014 | USA, Europe, Japan; male and female; mostly middle aged | olive oil, MUFA, MUFA:SFA, oleic acid, 6.5-30 years follow up | Lowest intake quantile group |
| Stroke | \| Lukas Schwingshackl et al. 2014 \| \| --- \| | USA, Europe, Japan; male and female; middle to old age | MUFA, olive oil,5.25-19.3 years follow up |  |

**Supplemental table5.** Characteristics of studies included in the blood lipids outcomes.

| **Outcomes** | **Study** | **Population** | **Intervention** | **Comparison** |
| --- | --- | --- | --- | --- |
| TG | Mojgan Morvaridzadeh et al. 2023 | Healthy individuals, hypercholesterolemia, coronary heart disease, type 2 diabetes, peripheral vascular disease patients, obese individuals. | EVOO,10-60ml/d,20%-30%TE | Palm oil, sunflower seed oil, soybean oil, corn oil, butter, refined olive oil, regular diet |
| TC | Mojgan Morvaridzadeh et al. 2023 |  |  |  |
| LDL | Mojgan Morvaridzadeh et al. 2023 |  |  |  |
| HDL | Mojgan Morvaridzadeh et al. 2023 |  |  |  |
| VLDL | Mojgan Morvaridzadeh et al. 2023 | Healthy individuals, hypercholesterolemia, type 2 diabetes | EVOO,54g/d,20%TE | Palm olein  Corn oil  Sunflower oil |
| ApoA-I | Mojgan Morvaridzadeh et al. 2023 | Healthy subjects, Hypercholesterolemia, High risk of ACVD, PVD, FM, Hypercholesterolemic (IIa) patients | EVOO,30-60ml/d,16-23%TE, 48-54g/d | Palm olein, Corn oil, soybean oil, refined olive oil, Cottonseed oil, regular diet |
| ApoB | Mojgan Morvaridzadeh et al. 2023 |  |  |  |
| lipid profile | Rosario Pastor et al. 2021 | Healthy population, overweight, metabolic syndrome, hypertension, hypercholesterolemia;18-over 70 years old, male and female | 4.5g OA; High-oleic sunflower oil(or capsule), High-oleic canola oil, 56g High-oleic peanuts, Olive oil, 200ml milk (3g OA), ground beef patties; 15-25% TE; 3-24w(mostly 4-12w) | PUFA; normal peanuts; SFA; Habitual Diet; Placebo |
| lipid profile | Rosario Pastor et al. 2021 | Non-alcoholic fatty liver disease (NAFLD),Dyslipidemia, Metabolic syndrome, Type 2 diabetes, Hypertension, Hypercholesterolemia patients  - | Olive oil liquid form(10-54g/d), capsule(2-4g/d); Mediterranean Diet with EVOO; olive oil for cooking; low fat diet with olive oil  - | PUFA, SFA, MUFA; supplement with Plant Sterols or Phospholipids or MCT Oil or CLA; low fat diet with high carbohydrate,regular diet  - |
| postprandial triglycerides over 8h | Milena Monfort-Pires et al.2016 | Healthy, normolipidemic individuals; hypertriglyceridemia; hyperlipoproteinemia; metabolic syndrome; Both lean and obese subjects. Sex:exclusively in men; a few included men and women; Mean age:21- 40 years or 19-65 years old | olive oil (often specified as refined or extra-virgin) or High-oleic sunflower oil; Type: shakes and solid mixed meals; 40-80g or 24%-83% | Primary Source:butter  , palm and cocoa oil, and high-palmitic sunflower oil; Dosage:  be isocaloric and isolipidic to the MUFA intervention |
| postprandial triglycerides over 4h | Milena Monfort-Pires et al.2016 |  |  |  |
| postprandial triglycerides over 6h | Milena Monfort-Pires et al.2016 |  |  |  |
| TC | Jabbarzadeh-Ganjeh, Bahareh et al.2023 | Healthy individuals, Hypercholesterolemia/Hyperlipidemia, Metabolic Syndrome, Overweight/Obesity, Coronary Artery Disease, NAFLD; Most studies included both sexes; young adults (~23 years) to older populations (~72 years) | Mostly EVOO, unspecific olive oil, Virgin Olive Oil, Refined Olive Oil; dosage: 15-89g/d (mostly 20-30g/d or 25-30g/d); 3 weeks to 156 weeks/3 years (mostly 4-12w); | Sunflower oil (most frequent comparator), rapeseed/canola oil, palm oil/palm olein, corn oil, soybean oil, flaxseed oil, butter or standard/usual diet |
| LDL | Jabbarzadeh-Ganjeh, Bahareh et al.2023 |  |  |  |
| HDL | Jabbarzadeh-Ganjeh, Bahareh et al.2023 |  |  |  |
| TAG | Jabbarzadeh-Ganjeh, Bahareh et al.2023 |  |  |  |

**Supplemental table5.** Characteristics of studies included in the all-cause mortality outcomes.

| **Outcomes** | **Study** | **Population** | **Intervention** | **Comparison** |
| --- | --- | --- | --- | --- |
| all-cause mortality | Miguel A. Martínez-Gonzalez et al. 2022 | EPIC,SUN,ENRICA,Three-City,BRHSNHS,HPFS, NIH-AARP, ICS, GISSI-Prevenzione,Ikaria,PREDIMED;adults and the elderly;healthy or disease | Unspecific Olive oil; FFQ;>7g/d->30g/d | Lowest olive oil intake; low fat diet |
| All-cause mortality | Lukas Schwingshackl et al. 2014 | USA, Europe, Japan, Australia; male and female; more than 35 years old | MUFA, MUFA:SFA, olive oil, 4-23 years follow up | Lowest intake quantile group |

**Supplemental table6.** Characteristics of studies included in the cancer outcomes.

| **Outcomes** | **Studies** | **Population** | **Intervention** | **Comparison** |
| --- | --- | --- | --- | --- |
| Cancer(incidence or mortality) | Miguel A. Martínez-Gonzalez et al. 2022 | EPIC, NHS, HPFS, NIH-AARP, E3N-EPIC cohort; PREDIMED | Unspecific Olive oil; Mediterranean Diet+EVOO; FFQ | Lowest olive oil intake; low fat diet |
| Breast cancer risk | Naria Sealy et al.2020 | Premenopausal or postmenopausal (18-85 years) women; in Mediterranean countries, Kuwait and Turkey | Dietary olive oil; times per week or gram/day | Highest vs Lowest OO intake |
| Gastrointestinal cancer | Christos Markellos et al.2022 | Gastrointestinal cancer patients (40-75 years) vs none-cancer patients or EPIC cohort, PREDIMED (35-80 years) | Unspecified OO type; Frequency or gram/day (FFQ) or quantile or continuous variable analysis | low or no olive oil consumption |
| Upper aerodigestive cancers | Christos Markellos et al.2022 | Upper aerodigestive cancers, most males with median age of 60 years | Unspecified OO type; frequency or gram/day (FFQ) | none-cancer patients with low or no olive oil consumption |
| Urinary tract cancers | Christos Markellos et al.2022 | Bladder cancer or urinary tract cancer male patients with a median age of 60 years | Unspecified Olive oil consumption (yes/no or quartiles) | benign urinary diseases or general population with no olive oil consumption |
| Colorectal cancer | Christos Markellos et al.2022 | Colorectal cancer patients both male and female (50-70 years) Vs none cancer or healthy population  or EPIC cohort with age above 50 years | Unspecified OO type; frequency or gram/day (FFQ) or continuous variable analysis | no olive oil consumption |
| Esophageal cancer | Christos Markellos et al.2022 | Male patients with esophageal squamous cell carcinoma, median age of 60 years | Unspecified OO type; frequency or gram/day (FFQ) | none-cancer patients with low or no olive oil consumption |
| Gastric cancer | Christos Markellos et al.2022 | Male and female gastric cancer patients(50-70years) | Unspecified OO type; frequency or yes or no (FFQ) | none-cancer patients with no olive oil consumption |
| Prostate cancer | Christos Markellos et al.2022 | Male prostate cancer (55-75years) | Unspecified OO type; frequency or yes or no (FFQ) | none-cancer patients or general population with low or no olive oil consumption |

**Supplemental table7.** Characteristics of studies included in the derma application outcomes.

| **Outcomes** | **Studies** | **Population** | **Intervention** | **Comparison** |
| --- | --- | --- | --- | --- |
| Incidence of RTOG grade 1–2 | Jolien Robijns et al.2023 | Nasopharyngeal carcinoma (stage III or IV) and breast cancer | Olive oil 3x/day or general skin care regimen + olive oil and calcium hydroxide emulsion 2x/day at the first day of RT and lasts 2 weeks post-RT | General skin care regimen and with placebo (water) |
| RTOG grade 2+ | Jolien Robijns et al.2023 |  |  |  |
| RTOG grade 3+ | Jolien Robijns et al.2023 |  |  |  |
| Incidence of Pressure Ulcers | Akram Hernández-Vásquez et al.2022 | ICU patients, immobilized patient, Nursing home residents at risk of PU onset (Braden Scale score< 14) | Mostly Extra virgin olive oil (liquid, spray, or cream); olive oil | HOFA solution or no moisturizing product |
| Adverse Events | Akram Hernández-Vásquez et al.2022 | ICU patients, immobilized patient, Nursing home residents at risk of PU onset (Braden Scale score< 14) | Mostly Extra virgin olive oil (liquid, spray, or cream) | HOFA solution |

| **Outcomes** | **Study** | **Population** | **Intervention** | **Comparation** |
| --- | --- | --- | --- | --- |
| maternal-fetal outcomes (SGA and LGA newborns, GDM, preeclampsia, and cardiovascular risk) | Anna Carolina Cortez-Ribeiro et al. 2023 | pregnant women with or without Gestational Diabetes Mellitus (GDM) | Mediterranean diet with EVOO or VOO, >40g/d or 50g/d or 36g/d, or FFQ | Mediterranean Diet restricted in fat or low-fat diet |
| cognitive fuction in the elder adults | Asra Fazlollahi et al. 2023 | The elderly aged 55 above; generally healthy, community-dwelling elderly, with high vascular/cardiovascular risk, Mild Cognitive Impairment, at risk for metabolic syndrome | Mediterranean Diet (MeDi)(+EVOO or nuts), 20-30g/d or 50ml/d, lasts 1year to 6.5 years, FFQ | low-fat diet or only Mediterranean Diet, levels of dietary exposure |
| hepatic steatosis | Yiwei Ma et al. 2023 | Primarily overweight/obese individuals (BMI >25-28 kg/m²) with a specific diagnosis of NAFLD or elevated IHL (>5%); mostly both sexes or only male; mean age ranged from 36 to 58 years | Extra Virgin Olive Oil (EVOO), Refined Olive Oil (ROO), and Olive Pomace Oil (OPO); 20-30 g/day; 8weeks-6month | Sunflower Oil (SFO), Rapeseed Oil (RA), Canola Oil (CO), or a control group with no oil supplement or standard care |
| liver enzymes | Yiwei Ma et al. 2023 | Primarily NAFLD, orPolycystic Ovarian Syndrome (PCOS) often associated with NAFLD; generally overweight/obese (BMI 25-40 kg/m²), mean age was similar, aged 29 to 46 years | Primarily Extra Virgin Olive Oil (EVOO) and standard Olive Oil (OO), commonly 20-30 g/day; Duration: 10 to 12 weeks. | Sunflower Oil (SFO), Canola Oil (CO), or a control group with no specific oil intervention |

**Supplemental table8.** Characteristics of studies included in the other outcomes.
